# Supplementary material for: Sirtuin2 blockade inhibits replication of human immunodeficiency virus-1 and Mycobacterium tuberculosis in macrophages and humanized mice
Source: Mol Ther. 2026 Mar 25;34(6):3535–47. doi: 10.1016/j.ymthe.2026.03.019 (PMC13239726; doi:10.1016/j.ymthe.2026.03.019)
Supplement: Document S1. Figures S1–S6 [file mmc1.pdf]

YMTHE, Volume *34*

Supplemental Information

Sirtuin2 blockade inhibits replication of human immunodeficiency virus-1 and *Mycobacterium tuberculosis* in macrophages and humanized mice

Vipul K. Singh, Abhishek Mishra, Khanghy Truong, Jose Alejandro Bohorquez, Suman Sharma, Arshad Khan, Franz Bracher, Kangling Zhang, Janice J. Endsley, Mark Endsley, Andrew P. Rice, Jason T. Kimata, Guohua Yi, and Chinnaswamy Jagannath

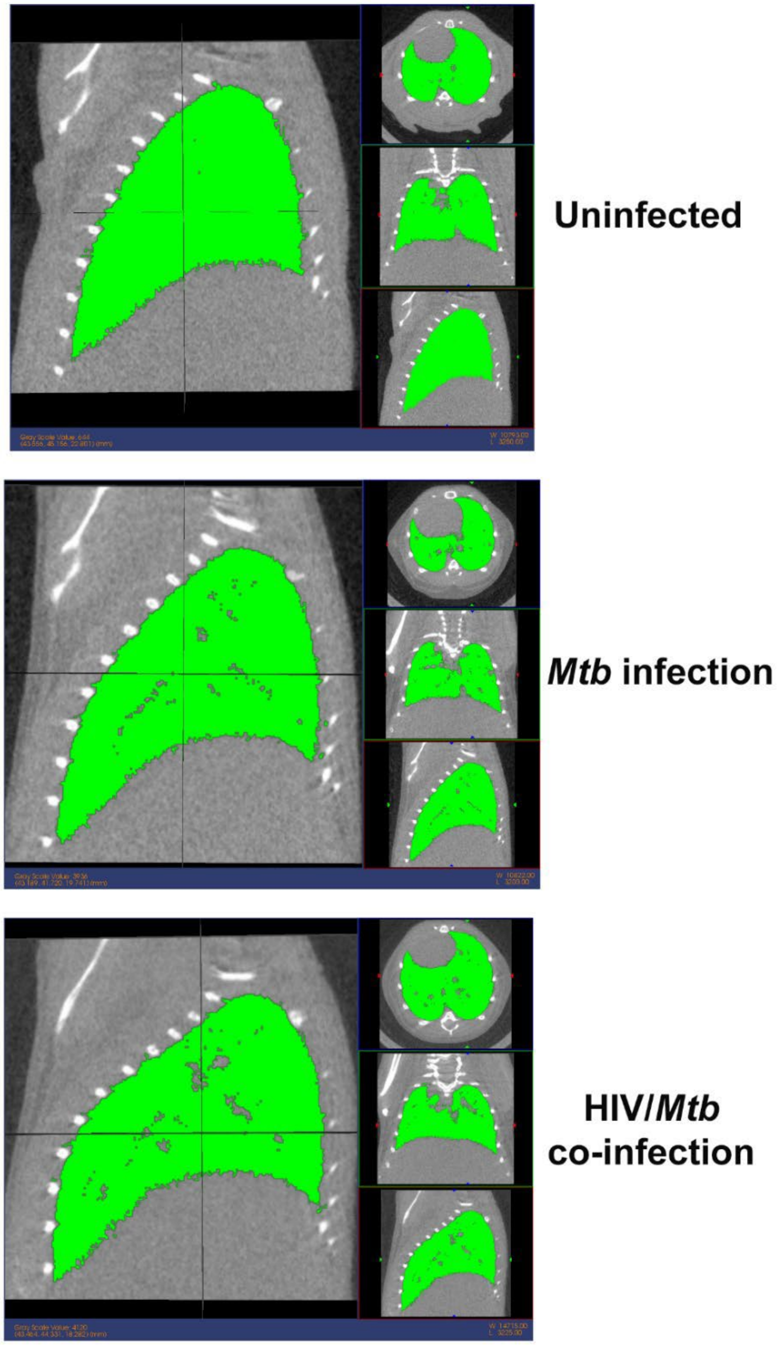

Figure S1: Representative lung volume pictures (green-filled) from animals in different experimental groups.

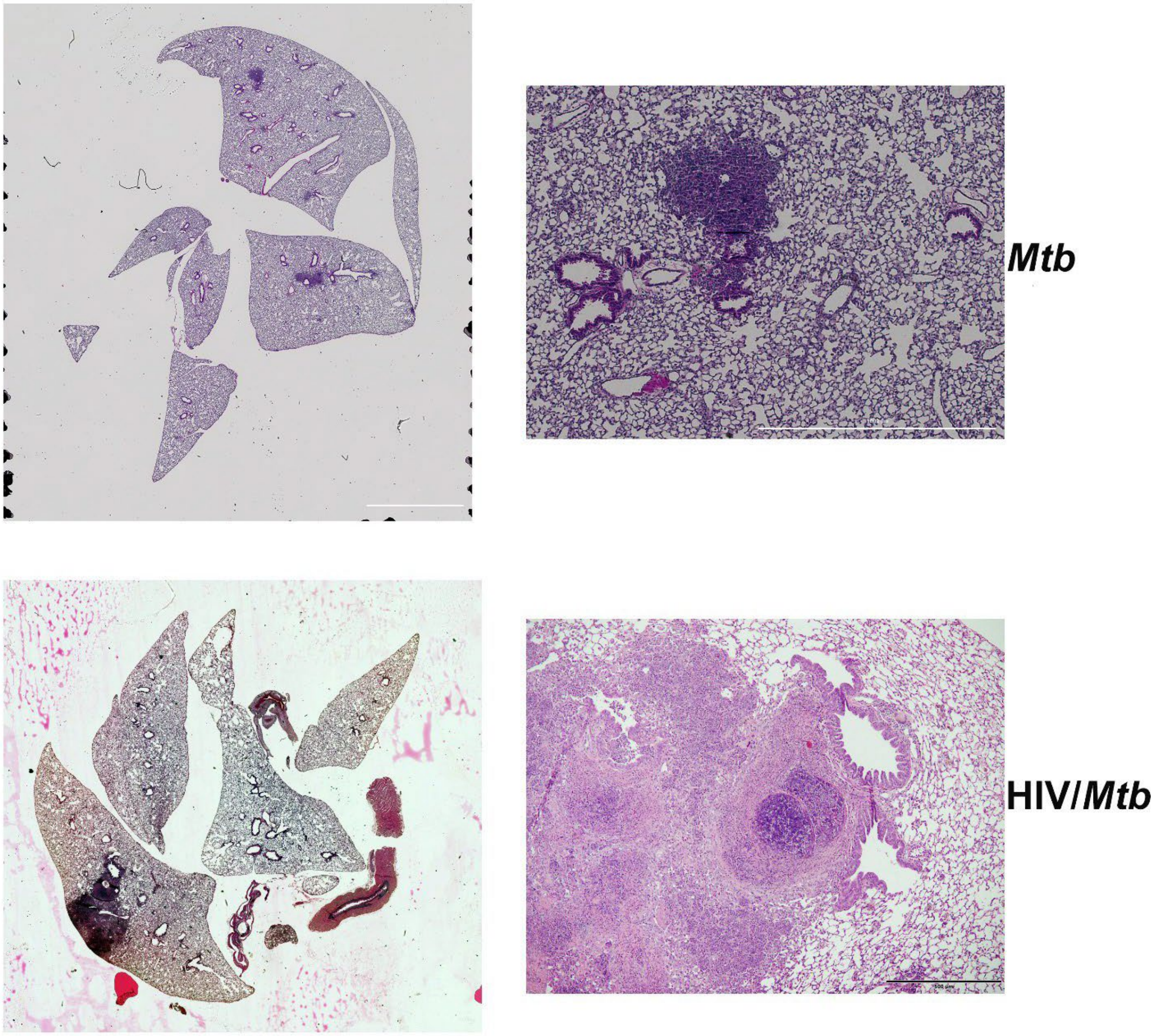

Figure S2: H&E staining of lung sections from representative *M.tuberculosis (Mtb)*-infected mouse and HIV/Mtb co-infected mouse show typical miliary TB pathology, caused by granuloma formation. The left panel shows the whole lungs, and the right panel shows typical granuloma structures in each group.

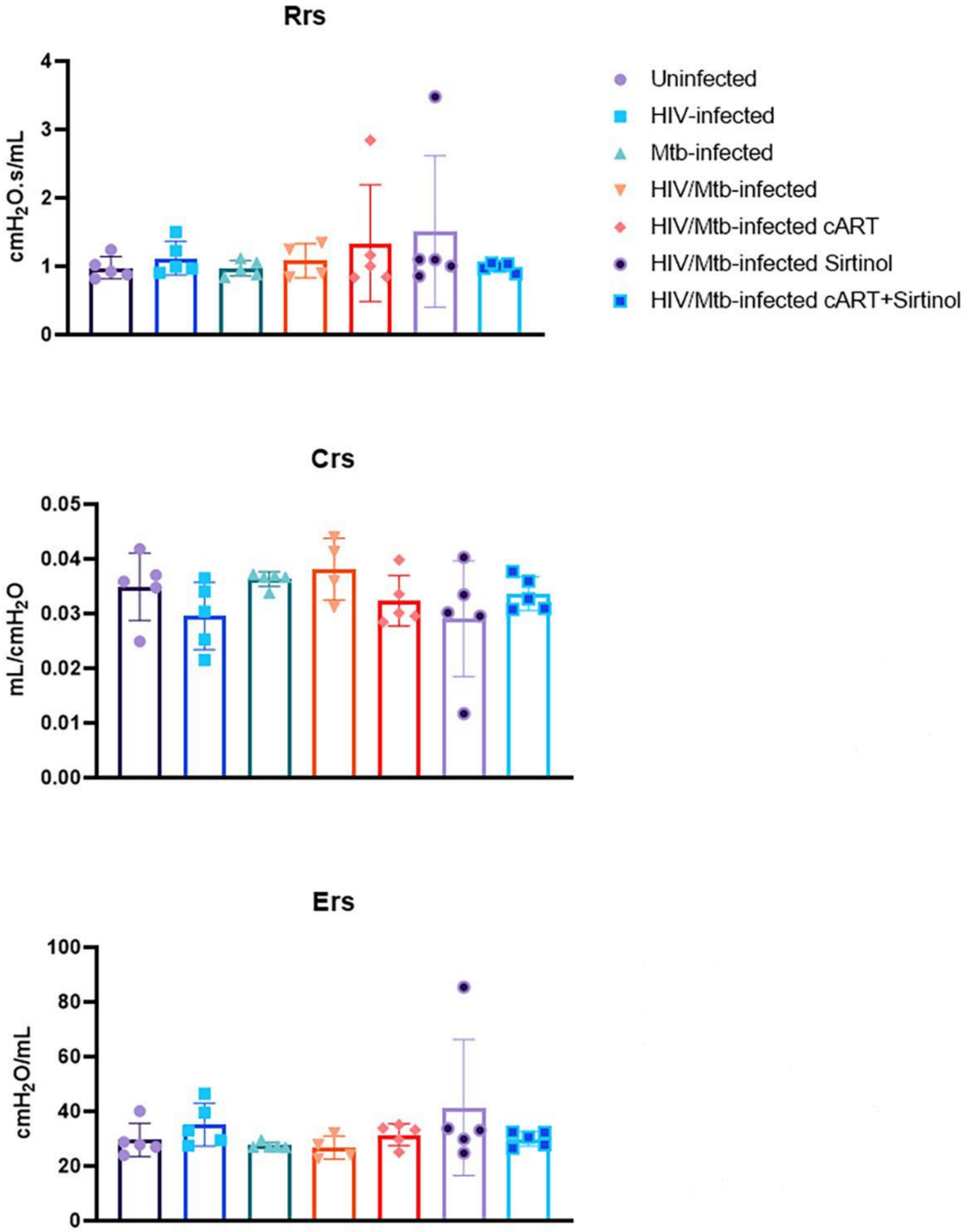

Figure S3: Pulmonary function test parameters: Resistance (Rrs), compliance (Crs) and elastance (Ers), were collected from animals in different treatment groups at the end of the experiment.

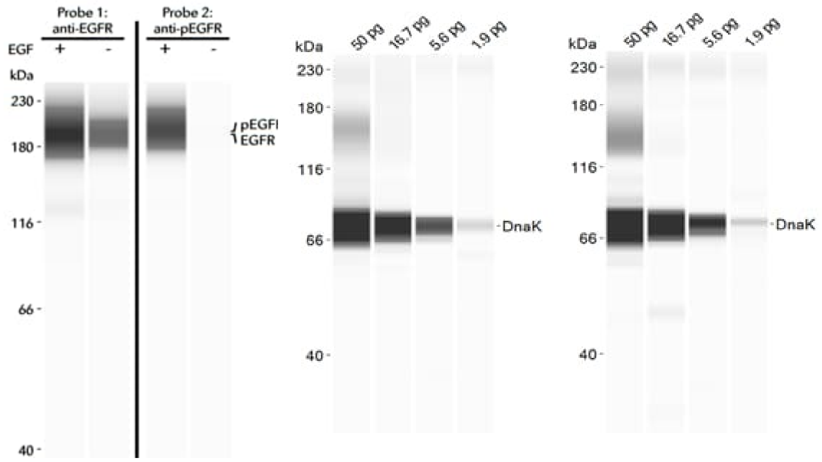

Figure S4: (Figure 2C) Illustration of capillary gel electrophoresis (samples are in individual lanes with markers; gels do not involve cutting and reproducing blots)

Abby's fully analyzed results can be viewed in either lane or electropherogram views. You can use the lane view option to compare band intensity or quantitatively analyze protein size and concentration. Compare the expression of proteins of interest to total protein. Data analysis with Compass's user-friendly software is quick and easy. Save graph and baseline options with run files and quickly annotate lane view results with Compass lane view features. *Lane annotation with Compass 6.0* saves hours spent annotating data in other programs. Abby is a trademark of ProteinSimple, Corp.(blots are reproduced from Abby website.

Name	Signal	Actin	Area	Bkgnd.	Type	Image Na	Channel	Name	Signal	ReA1	Area	Bkgnd.	Type	Ratio	Ratio	Average	STDV	Name	Average		
1	31	35.3	1410	0.00306	Signal	0001692_	800	25	0.0323	0.103	1334	5.29E-05	Signal	0.00292	0.0023	0.0026	0.0004	M0	0.00263	1	
2	39.8	44	1628	0.00257	Signal	0001692_	800	26	0.0386	0.105	1271	5.23E-05	Signal	0.00239	0.0022	0.0023	0.0001	M1	0.00232	1	
3	54.1	58.1	1462	0.00276	Signal	0001692_	800	27	0.0553	0.115	1131	5.26E-05	Signal	0.00198	0.0017	0.0019	0.0002	M2	0.00186	1	
4	18.1	22.1	1088	0.00373	Signal	0001692_	800	28	0.0297	0.0755	864	0.000053	Signal	0.00342	0.0033	0.0033	0.0001	M0+R	0.00334	1.26939	
5	27.3	31.4	1400	0.00291	Signal	0001692_	800	29	0.037	0.0929	1064	5.25E-05	Signal	0.00296	0.0029	0.0029	0.0000	M1+R	0.00292	1.26175	
6	25.2	29.4	1160	0.00361	Signal	0001692_	800	30	0.0357	0.0962	1200	5.04E-05	Signal	0.00327	0.0028	0.0030	0.0004	M2+R	0.00302	1.619351	
7	5.03	7.03	650	0.00308	Signal	0001692_	800	31	0.00479	0.035	616	4.91E-05	Signal	0.00498	0.0057	0.0053	0.0005	M0+S	0.00533	2.024864	
8	24.7	28.1	1073	0.00318	Signal	0001692_	800	32	0.0354	0.0881	1064	4.95E-05	Signal	0.00314	0.0028	0.0030	0.0002	M1+S	0.00299	1.291252	
9	11	14.6	1170	0.00305	Signal	0001692_	800	33	0.0398	0.0943	1073	5.08E-05	Signal	0.00646	0.0062	0.0063	0.0002	M2+S	0.00631	3.386049	
10	23.7	27.5	1295	0.00299	Signal	0001692_	800	34	0.0456	0.0995	1064	5.07E-05	Signal	0.00362	0.0034	0.0035	0.0002	M0+R+S	0.00349	1.324758	
11	4.84	7.14	900	0.00256	Signal	0001692_	800	35	0.00462	0.0438	825	4.75E-05	Signal	0.00613	0.0079	0.0070	0.0012	M1+R+S	0.00699	3.01784	
12	0.615	3.62	1326	0.00227	Signal	0001692_	800	36	0.00323	0.0448	880	4.73E-05	Signal	0.01238	0.0197	0.0160	0.0052	M2+R+S	0.01602	8.604058	
13	34.4	38.5	1440	0.00282	Signal	0001692_	800	37	0.042	0.129	1680	5.18E-05	Signal	0.00335	0.0028	0.0031	0.0004	M0+HIV	0.00307	1.166172	1
14	42	46	1496	0.00268	Signal	0001692_	800	38	0.0523	0.126	1386	5.28E-05	Signal	0.00274	0.0029	0.0028	0.0001	M1+HIV	0.00280	1.06568	1
15	41.2	45.1	1440	0.00269	Signal	0001692_	800	39	0.0605	0.131	1320	5.36E-05	Signal	0.00290	0.0029	0.0029	0.0000	M2+HIV	0.00293	1.571681	1
16	58	62.1	1386	0.00301	Signal	0001692_	800	40	0.0459	0.12	1320	5.64E-05	Signal	0.00193	0.0020	0.0020	0.0000	M0+R+HI\	0.00197		0.640758
17	43.5	47.4	1365	0.00282	Signal	0001692_	800	41	0.0507	0.113	1147	5.41E-05	Signal	0.00238	0.0028	0.0026	0.0003	M1+R+HI\	0.00261		0.931704
18	60.3	64.6	1505	0.00286	Signal	0001692_	800	42	0.0727	0.139	1240	5.36E-05	Signal	0.00215	0.0025	0.0023	0.0002	M2+R+HI\	0.00232		0.792019
19	18.5	21.6	1188	0.00262	Signal	0001692_	800	43	0.0497	0.109	1140	5.19E-05	Signal	0.00505	0.0052	0.0051	0.0001	M0+S+HIV	0.00514		1.676288
20	13.6	16.4	1085	0.00257	Signal	0001692_	800	44	0.051	0.114	1200	5.22E-05	Signal	0.00695	0.0070	0.0070	0.0000	M1+S+HIV	0.00698		2.489035
21	8.82	11.8	986	0.00299	Signal	0001692_	800	45	0.0505	0.113	1209	5.14E-05	Signal	0.00958	0.0096	0.0096	0.0000	M2+S+HIV	0.00958		3.271632
22	3.94	6.4	910	0.00271	Signal	0001692_	800	46	0.0334	0.0906	1102	5.19E-05	Signal	0.01416	0.0131	0.0136	0.0007	M0+R+S+ł	0.01363		4.443268
23	26.5	29.6	1260	0.00249	Signal	0001692_	800	47	0.081	0.158	1344	5.71E-05	Signal	0.00534	0.0055	0.0054	0.0001	M1+R+S+ł	0.00542		1.931691
24	14.5	17.9	1591	0.00211	Signal	0001692_	800	48	0.0469	0.114	1353	4.95E-05	Signal	0.00637	0.0063	0.0063	0.0001	M2+R+S+ł	0.00633		2.162345

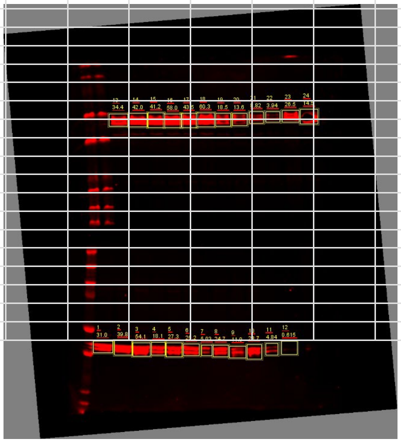

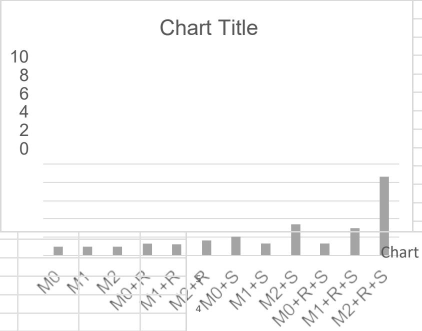

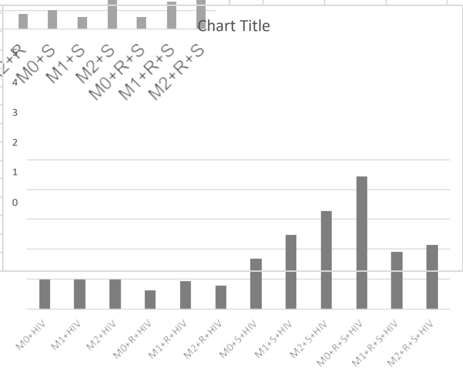

Group	Sample name	absorband	concentration (µg/mL)	Sample µ	Dye µl	lysis buffe
Add Res and Sirtinol and tinfectd with HIV	#1, M0_HIV	0.271	1648	24.3	15	20.7
	#2, M1_HIV	0.2334	1272	31.4	15	13.6
	#3, M2_HIV	0.2516	1454	27.5	15	17.5
	#4, M0_None	0.2199	1137	35.2	15	9.8
	#5, M1_None	0.2139	1077	37.1	15	7.9
	#6, M2_None	0.205	988	40.5	15	4.5
	#7, M0_Res	0.1759	697	57.4	15	-12.4
	#8, M1_Res	0.1885	823	48.6	15	-3.6
	#9, M2_Res	0.2008	946	42.3	15	2.7
	#10, M0_Sirtinol	0.1931	869	46.0	15	-1.0
	#11, M1_Sirtinol	0.2643	1581	25.3	15	19.7
	#12, M2_Sirtinol	0.1984	922	43.4	15	1.6
	#13, M0_HIV+Res	0.2157	1095	36.5	15	8.5
	#14, M1_HIV+Res	0.2216	1154	34.7	15	10.3
	#15, M2_HIV+Res	0.2234	1172	34.1	15	10.9
	#16, M0_HIV+Sirtinol	0.2261	1199	33.4	15	11.6
	#17, M1_HIV+Sirtinol	0.2399	1337	29.9	15	15.1
	#18, M2_HIV+Sirtinol	0.2911	1849	21.6	15	23.4
	#19, M0_HIV+Res+Sirtinol	0.2518	1456	27.5	15	17.5
	#20, M1_HIV+Res+Sirtinol	0.1508	446	89.7	15	-44.7
	#21, M2_HIV+Res+Sirtinol	0.1971	909	44.0	15	1.0
	#22, M0_Res+Sirtinol	0.1206	144	277.8	15	-232.8
	#23, M1_Res+Sirtinol	0.1396	334	119.8	15	-74.8
	#24, M2_Res+Sirtinol	0.1198	136	294.1	15	-249.1
Infected with HIV for 3d then add drugs for 2d	#25, M0_HIV	0.2515	1453	27.5	15	17.5
	#26, M1_HIV	0.2067	1005	39.8	15	5.2
	#27, M2_HIV	0.2585	1523	26.3	15	18.7
	#28, M0_None	0.1841	779	51.3	15	-6.3
	#29, M1_None	0.1705	643	62.2	15	-17.2
	#30, M2_None	0.174	678	59.0	15	-14.0
	#31, M0_Res	0.2322	1260	31.7	15	13.3
	#32, M1_Res	0.2302	1240	32.3	15	12.7
	#33, M2_Res	0.2012	950	42.1	15	2.9
	#34, M0_Sirtinol	0.2392	1330	30.1	15	14.9
	#35, M1_Sirtinol	0.2309	1247	32.1	15	12.9
	#36, M2_Sirtinol	0.2216	1154	34.7	15	10.3
	#37, M0_HIV+Res	0.2376	1314	30.4	15	14.6
	#38, M1_HIV+Res	0.2929	1867	21.4	15	23.6
	#39, M2_HIV+Res	0.2807	1745	22.9	15	22.1
	#40, M0_HIV+Sirtinol	0.2393	1331	30.1	15	14.9
	#41, M1_HIV+Sirtinol	0.2207	1145	34.9	15	10.1
	#42, M2_HIV+Sirtinol	0.2667	1605	24.9	15	20.1
	#43, M0_HIV+Res+Sirtinol	0.2454	1392	28.7	15	16.3
	#44, M1_HIV+Res+Sirtinol	0.1701	639	62.6	15	-17.6
	#45, M2_HIV+Res+Sirtinol	0.151	448	89.3	15	-44.3
	#46, M0_Res+Sirtinol	0.2155	1093	36.6	15	8.4
	#47, M1_Res+Sirtinol	0.2399	1337	29.9	15	15.1
	#48, M2_Res+Sirtinol	0.1999	937	42.7	15	2.3

Figure S5: Main text Figure 4: Histone acetylation raw data set and sample information.

Raw data sets for Figures 6A (CD4 and CD8 ratio):

Pre-challenge	15 dpi (HIV)/7 dpi (Mtb)	28 dpi (HIV)/21 dpi (Mtb)	35 dpi (HIV)/28 dpi (Mtb)
3.35	0.076852	0.245732	0.555085
3.16	0.11768	0.338776	0.438735
36		10.01346	8.587963
3.44	1.625	0.9	1.72
1.83	0.009634	0.358098	0.706004

Raw data for Figures 6B (HIV viral load):

Pre-challenge	15 dpi (HIV)/7 dpi (Mtb)	28 dpi (HIV)/21 dpi (Mtb)	35 dpi (HIV)/28 dpi (Mtb)
1	1	13137023.09	1.2E+08
1	1	1	1
1	1	1	1
1	1	30390881.28	1.93E+08
1	1	154940081.7	67910680

Raw data for Figures 6C (Lungs and spleen Mtb CFUs)

	Lung			Spleen
Initial infection	Mtb Control	HIV-Mtb Co-infection	Mtb Control	HIV-Mtb Co-infection
50	124000	680000	5200	5400
30	560000	2000000	8600	41600
270	140000	208000	4600	40000
	86000	10400	3800	187200
	356000		15800	800
	160000		1200	
	736000		10000	
	396000		10000	
	466000		12000	

Raw data for Figures 6D (PET assay)

Rrs			Crs			Ers			Lung volume		
Uninfected	MtbControl	Mtbcoinfected	Uninfected	Mtbcontrol	Mtbcoinfected	Uninfected	Mtbcontrol	Mtbcoinfected	Uninfected	Mtbcontrol	Mtbcoinfected
0.869	2.282222	3.745	0.056	0.171306	0.011	17.914	5.839117	88.718	1260	711.33	637.7
0.78	7.870395	0.972	0.053	0.024731	0.036	18.884	40.43537	27.515	1290	1300	934.51
0.791	2.245803	1.182	0.055	0.012605	0.048	18.03	79.33481	20.721	1310	830.6	1070
	1.089791	0.897		0.032	0.047		31.411	21.079		1290	1190
	1.742787	0.881		0.014918	0.047		67.04054	21.338		1040	1170
	0.913262	3.642081		0.037181	0.011638		26.8992	86.05211		1510	1270
	1.088283	0.97224		0.032174	0.036345		31.08131	27.51465		1010	1110
		1.181838			0.048261			20.72088			1060
		0.896671			0.047441			21.07897			1150
		0.881023			0.046865			21.33839			

Figure S6: Raw data sets for Figure S6A-D.
