# Supplementary material for: Sirtuin2 blockade inhibits replication of human immunodeficiency virus-1 and Mycobacterium tuberculosis in macrophages and humanized mice
Source: Mol Ther. 2026 Mar 25;34(6):3535–47. doi: 10.1016/j.ymthe.2026.03.019 (PMC13239726; doi:10.1016/j.ymthe.2026.03.019)
Supplement: Document S2. Article plus supplemental information [file mmc2.pdf]

Molecular Therapy
Original Article

Sirtuin2 blockade inhibits replication of human immunodeficiency virus-1 and *Mycobacterium tuberculosis* in macrophages and humanized mice

Vipul K. Singh,[1,7] Abhishek Mishra,[1,7] Khanghy Truong,[2,7] Jose Alejandro Bohorquez,[3,7] Suman Sharma,[2] Arshad Khan,[1] Franz Bracher,[4] Kangling Zhang,[5] Janice J. Endsley,[6] Mark Endsley,[6] Andrew P. Rice,[2] Jason T. Kimata,[2] Guohua Yi,[3] and Chinnaswamy Jagannath[1]

[1]Department of Pathology and Genomic Medicine, Houston Methodist Research Institute, Weill Cornell Medicine, Houston, TX, USA; [2]Department of Molecular Virology & Microbiology, Baylor College of Medicine, Houston, TX, USA; [3]Department of Cellular and Molecular Biology, The University of Texas Health Science Center at Tyler, Tyler, TX, USA; [4]Department of Pharmacy, Ludwig-Maximilians University, Munich, Germany; [5]Department of Pharmacology and Microbiology and Immunology, UTMB Galveston, Galveston, TX, USA; [6]Microbiology and Immunology, UTMB Galveston, Galveston, TX, USA

Co-infections with *Mycobacterium tuberculosis* (Mtb) and human immunodeficiency virus-1 (HIV-1) present a critical health challenge and require treatment for patient survival. We found that human M1 macrophages inhibit Mtb growth, while M2 macrophages, characterized by elevated Sirt2 expression, permit Mtb growth. Further, we found that HIV-1 augmented Sirt2 gene expression in MΦs. Therefore, we explored the therapeutic potential of sirtuin-modulating drugs using human MΦs. Sirtinol, a Sirt2 inhibitor, significantly reduced HIV-1 growth in M0, M1, and M2-MΦs by >1log10 over 7 days. Conversely, individual doses of resveratrol and SRT1460, which activate Sirt1, did not affect HIV-1. However, their combination showed a strong synergistic inhibition of HIV-1. The combination of sirtinol with resveratrol was neither synergistic nor antagonistic. Sirtinol upregulated *iNOS* and *ATG5* mRNA in HIV-1-infected MΦs in a phenotype-dependent manner. In a humanized mouse model (Hu-NSG-SGM3) co-infected with Mtb H37Rv and the HIV-1 BAL strain, treatment with sirtinol alone, or in combination with combination antiretroviral therapy (cART), showed promising results; sirtinol alone reduced Mtb growth, while its combination with cART effectively inhibited HIV-1 replication in the organs. We propose that Sirt2 blockade and Sirt1-activation represent a novel dual therapeutic strategy for treating HIV-1 and Mtb co-infections.

INTRODUCTION

Tuberculosis is the second leading cause of death from infectious diseases, claiming ∼1.2 million lives yearly. An unusual feature of tuberculosis is that the causative pathogen, *Mycobacterium tuberculosis* (Mtb), can either rapidly progress to active lung infection in ∼10% of infected individuals or remain dormant for decades in ∼90%. Human immunodeficiency virus-1 (HIV-1) is a similarly devastating infectious agent. Importantly, one-fourth of the human population is latently infected with Mtb (LTBI), and these individuals serve as a reservoir for potential tuberculosis reactivation. This risk is exacerbated as HIV-1 progressively depletes CD4 T cells, which are critical for anti-tuberculosis immunity. Curiously, HIV-1-infected individuals remain susceptible to tuberculosis even when CD4 T cell counts are within normal ranges, suggesting that myeloid/macrophages (MΦ) may play a role during tuberculosis/HIV-1 co-infection. Despite the hostile environment of MΦs, Mtb can survive, and HIV-1 can replicate within them, often providing a latent reservoir.[1,2] The lethal nature of untreated co-infections underscores the urgent need for a better understanding of the molecular interplay between Mtb and HIV-1 within MΦs.

Most studies involving Mtb and HIV-1 have used undifferentiated, naive MΦs. We recently reported functional heterogeneity between human M1- and M2-MΦs compared with naive MΦs (M0-MΦs) and revealed differences in pro-inflammatory and anti-inflammatory cytokine production correlating with different transcriptomes.[3] IFN-γ-stimulated M1-MΦs eliminate Mtb through autophagy and nitric oxide (NO)-dependent mechanisms, whereas IL-4-stimulated M2-MΦs permit Mtb growth due to decreased autophagy and NO levels. Importantly, Mtb-infected M1- and M2-MΦs exhibit differential induction of NAD^+-dependent sirtuin-type histone deacetylases. Blockade of Sirtuin2 (Sirt2) using sirtinol polarizes naive or M2-MΦs to M1-MΦs, which can eliminate Mtb. Intriguingly, Sirt2

Received 29 November 2024; accepted 17 March 2026;
https://doi.org/10.1016/j.ymthe.2026.03.019.

[7]These authors contributed equally

Correspondence: Jason Kimata, Department of Molecular Virology & Microbiology, Baylor College of Medicine, Houston, TX, USA.
E-mail: jkimata@bcm.edu
Correspondence: Guohua Yi, Department of Cellular and Molecular Biology, The University of Texas Health Science Center at Tyler, Tyler, TX, USA.
E-mail: guohua.yi@uttyler.edu
Correspondence: Chinnaswamy Jagannath, Department of Pathology and Genomic Medicine, Houston Methodist Research Institute, Weill Cornell Medicine, Houston, TX, USA.
E-mail: cjagannath@houstonmethodist.org

Published by Elsevier Inc. on behalf of The American Society of Gene and Cell Therapy.
This is an open access article under the CC BY license (http://creativecommons.org/licenses/by/4.0/).

blockade also inhibits HIV-1 growth in human MΦs, irrespective of their M0, M1, or M2 phenotype. Thus, sirtuins appear to be critical epigenetic modifiers of the early interactions between Mtb and HIV-1 in MΦs.

Many studies indicate that Mtb and HIV-1 exacerbate each other's effects; therefore, we hypothesized that Sirt2 activation by Mtb impairs MΦ function, thereby facilitating HIV-1 replication. Intriguingly, the HIV-1 transactivator protein Tat binds to and inhibits Sirt1 histone deacetylase, increasing the acetylation of the NF-kB p50/p65 subunit, which in turn leads to T cell hyperactivation and increased HIV-1 replication.[4,5] Therefore, prior infection with HIV-1 may reduce Sirt1 levels in MΦs, increasing Mtb growth.[6,7] Multiple sirtuin inhibitor drugs have been evaluated for chemotherapy.[8] In this study, we report that Sirt2 inhibits the replication of HIV1 and Mtb in human MΦs and is a promising target for developing immunochemotherapy for treating co-infections.

RESULTS

Sirtuin2 blockade inhibits Mtb growth in human macrophages and mice

We recently reported that human MΦs exhibit differential responses to Mtb infection, influenced by sirtuin expression levels. Specifically, in IFN-γ (M1) and IL-4 (M2) pre-programmed and rested MΦs that were subsequently infected with Mtb, we found that Sirt5^{hi} M1-MΦs restricted Mtb through autophagy and NO production. In contrast, Sirt2^{hi} M2-MΦs permitted Mtb growth due to reduced autophagy and NO levels.[3] Notably, Mtb infection upregulated transcripts for additional sirtuins in both M1- and M2-MΦs (Figure 1A). Further, infecting naive M0-MΦs using virulent Mtb (but not the attenuated BCG vaccine) progressively increased Sirt2 gene expression (Figure 1B). PCR assays conducted earlier showed elevated Sirt2 levels in peripheral blood mononuclear cells (PBMCs) from patients with active tuberculosis compared with healthy contacts, who showed an upregulation of Sirt5.[3] These findings suggest that Sirt2 is a druggable target for Mtb control in human MΦs. Figure 1C shows that Sirt2 blockade using sirtinol, but not the Sirt5 inhibitor balsalazide, inhibited Mtb growth in naive MΦs. Sirt1 inhibition was also ineffective, consistent with reports that Sirt1 activation might suppress Mtb growth in MΦs.[9] To determine the role of Sirt2 *in vivo*, C57BL/6 mice were aerosol-infected using Mtb Erdman, followed by sirtinol treatment. Sirtinol treatment significantly reduced bacterial growth in the lungs (Figure 1D). Together, these data indicate that targeting Sirt2 with sirtinol is an effective strategy for controlling tuberculosis in both MΦs and mouse models.

HIV-1 infection induces sirtuins in human macrophages

Because the HIV-1 transactivator Tat protein binds and inhibits Sirt1 that increases HIV-1 replication,[4,5] we hypothesized that it may also induce Sirt2 in MΦs. Using donor-derived MΦs, we identified that, as early as 18 h post infection, HIV-1 increased the levels of mRNA transcripts for Sirt1, Sirt2, and Sirt3 in both M0- and M1-MΦs, whereas M2-MΦs displayed a relatively reduced response to infection (Figure 2A). Sirt1-7 regulates multiple functions in mammalian cells, and our previous research has shown that blockade of one sirtuin can enhance the gene expression of others. Given the prominent roles of Sirt1 and Sirt2, we treated HIV-1-infected MΦs with the Sirt1 activator resveratrol and the Sirt2 inhibitor sirtinol. Subsequent PCR analysis of sirtuins revealed that inhibiting Sirt2 with sirtinol resulted in increased expression of Sirt5, 6, and 7 in M0-MΦs, suggesting a cross-regulation among sirtuins upon Sirt2 blockade. Sirtuins can also influence autophagy and NO-dependent antimicrobial mechanisms in MΦs.[10,11] Figures 2B and 2C illustrate that Sirt2 blockade upregulated mRNA transcripts for autophagy (*ATG5*) and NO production (*iNOS*) biomarkers in MΦs. However, iNOS protein levels decreased after Sirt2 blockade in HIV-infected MΦs for which we have no clear explanation; perhaps HIV-1 interferes with the translation of *iNOS* gene.[12,13]

Sirtuin2 blockade inhibits HIV-1 replication in human macrophages

Our data indicate that sirtuins, including Sirt1 and Sirt2, differentially regulate antimicrobial functions and are therapeutic targets for HIV-1 infection. Because treating infection and co-infection is difficult, and often requires multiple drugs, we first examined the effects of Sirt1 activation (due to the interaction of HIV-1 Tat with Sirt1) and Sirt2 blockade on HIV-1 replication. We observed a dose-dependent effect of noncytotoxic doses of Sirt1 activators (resveratrol, 20 μM; SRT1460, 3 μM) and inhibitors of Sirt2 (sirtinol, 38 μM), Sirt3 (3-TYP; 377 μM), and Sirt5 (balsalazide; 10 μM) to inhibit HIV-1. When administered as a single drug dose, 24 h post HIV-1 infection, only sirtinol, a Sirt2 inhibitor, showed a significant 2log10 reduction in HIV-1 replication in MΦs (Figure 3A). Conversely, resveratrol (a Sirt1 activator) and balsalazide (a Sirt5 inhibitor) also reduced HIV-1 replication, but to a lesser extent (1log10 between days 3 and 7). In two separate experiments, we observed that HIV-1 replication resumed after it was initially drastically reduced by sirtinol (Figure 3A). Therefore, in three separately conducted experiments using different sets of donor MΦs, drugs were administered on day 1 post infection and re-applied on days 3 and 5. Remarkably, sirtinol consistently inhibited HIV1 replication (a 2log10 reduction at day 3 post infection and 3log10 decrease by days 5 and 7) (Figure 3B). Intriguingly, neither resveratrol nor SRT1460 (Sirt1-activator) alone inhibited HIV-1, although their combined use resulted in a synergistic effect, reducing HIV1 replication by 1–2log10. A combination of sirtinol and resveratrol was also effective, suggesting that resveratrol is not antagonistic to sirtinol. MΦ viability remained >90% in these assays. Although higher doses of sirtuin-modulating drugs were not evaluated, these data suggest that Sirt1 activation and Sirt2 blockade are effective strategies for controlling HIV-1 replication in MΦs.

Sirt2 inhibition with sirtinol and Sirt1 activation with resveratrol increases Rel-A/NF-kB acetylation

A major regulatory mechanism during microbial infection is the acetylation and methylation of histones and enzymes that regulate the expression of immunity-related genes and the polarization of MΦs.[14] In the context of HIV-1, NAD-independent HDACs regulate

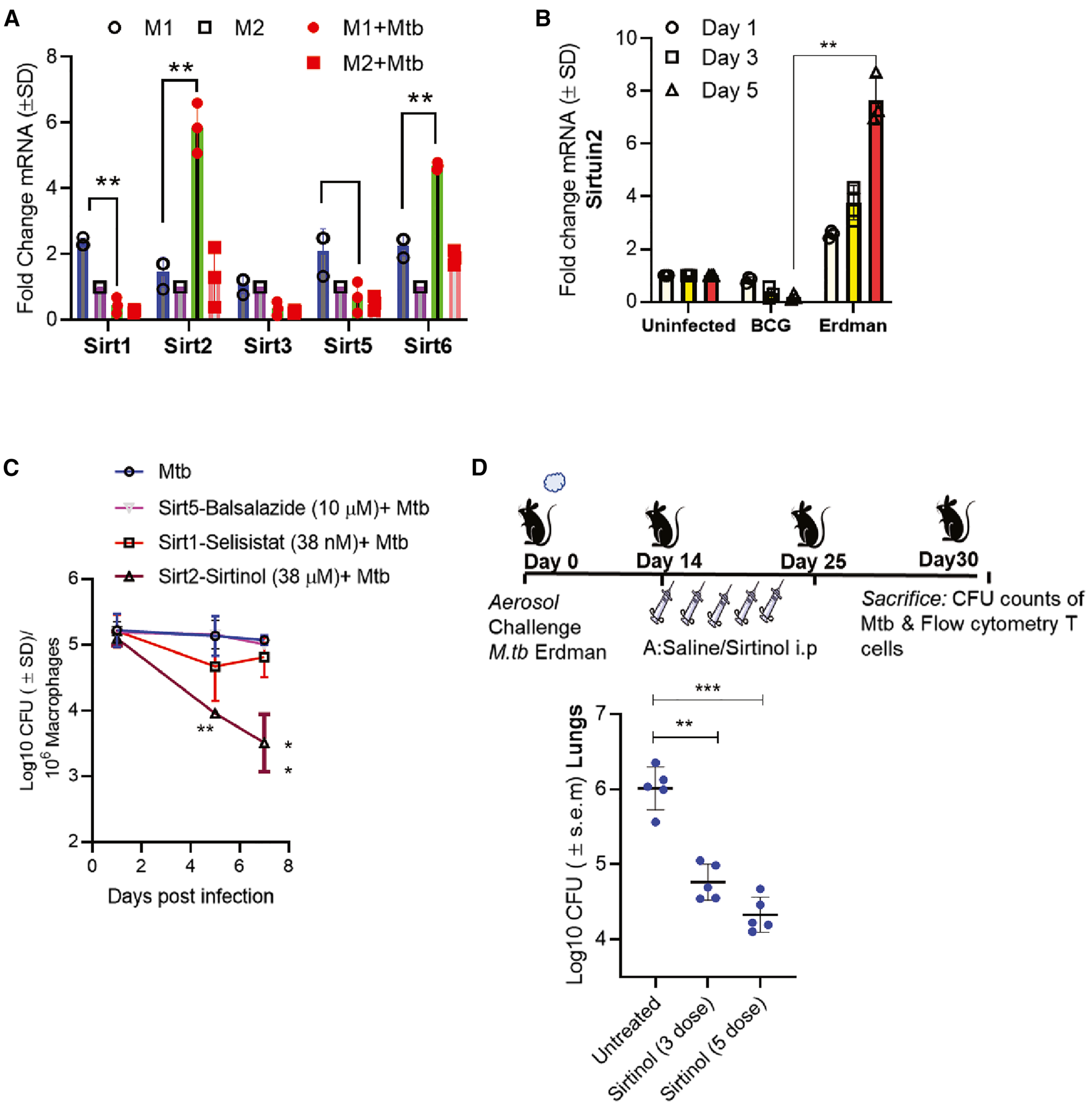

Figure 1. Sirtuin2 histone deacetylase regulates *Mycobacterium tuberculosis* growth in human macrophages and C57BL/6 mice

$CD14^+$ bead-purified macrophages derived from healthy donors (MΦs; pooled from 3 individuals) were either left naive (M0) or differentiated into M1-MΦs with IFN-γ or M2-MΦs with IL-4 and then rested and processed as follows. (A) M1 and M2-MΦs were infected with Mtb Erdman (MOI = 1) for 4 h, washed, and incubated, and Trizol lysates from triplicate wells collected 24 h post infection were assessed for mRNA expression of sirtuins as indicated (** $p < 0.009$; two-tailed t test). (B) Pooled MΦs ($n = 3$ donors per pool; 2 pools) were infected with either the BCG vaccine or Mtb (MOI = 1), washed, and incubated, and Trizol lysates were tested for mRNA expression of Sirt2 (** $p < 0.009$; two-tailed t test). (C) Pooled MΦs ($n = 3$ donors per pool; 2 pools) infected with Mtb were cultured and then treated with the indicated drugs (sirtinol for sirtuin2 inhibition at 38 μM; selisistat for sirtuin1 inhibition at 38 nM; balsalazide for sirtuin5 inhibition at 10 μM), followed by a colony forming unit (CFU) assay (** to measure Mtb growth; ** $p < 0.005$; one-way ANOVA). (D) Age and sex-matched C57BL/6 mice ($n = 5$ per group, 4–8 weeks, male + female) were infected with Mtb H37Rv (100 CFU per mouse) using a Glas-Col aerosol chamber. On days indicated post infection, mice were injected intraperitoneally with 10 mg sirtinol/kg body weight. Mice were sacrificed on day 30 post infection to assess Mtb CFU counts in the lungs by plating homogenates on 7H11agar (** $p < 0.007$; *** $p < 0.006$; two-way ANOVA with Tukey's posttest).

viral gene expression, and HDAC inhibitors have been used for therapy. Compounds like entinostat reactivate latent HIV-1 and induce maximal HIV-1 protein expression when used in combination with bryostatin-1, which is a protein kinase C agonist.[15] Given the effectiveness of Sirt1 activation and Sirt2 blockade against HIV-1 (Figure 3), we used donor-derived MΦs, either as naive M0 or differentiated into M1 or M2, rested them, and then infected them with HIV-1, following the earlier described drug treatment protocol (Figure 3). Cell lysates collected on day 4 post drug treatment were analyzed for Rel-A acetylation using multi-OMICS approaches.[16] Figure 4 shows that both sirtinol and resveratrol increased Rel-A acetylation compared with levels observed in HIV-1-infected MΦs

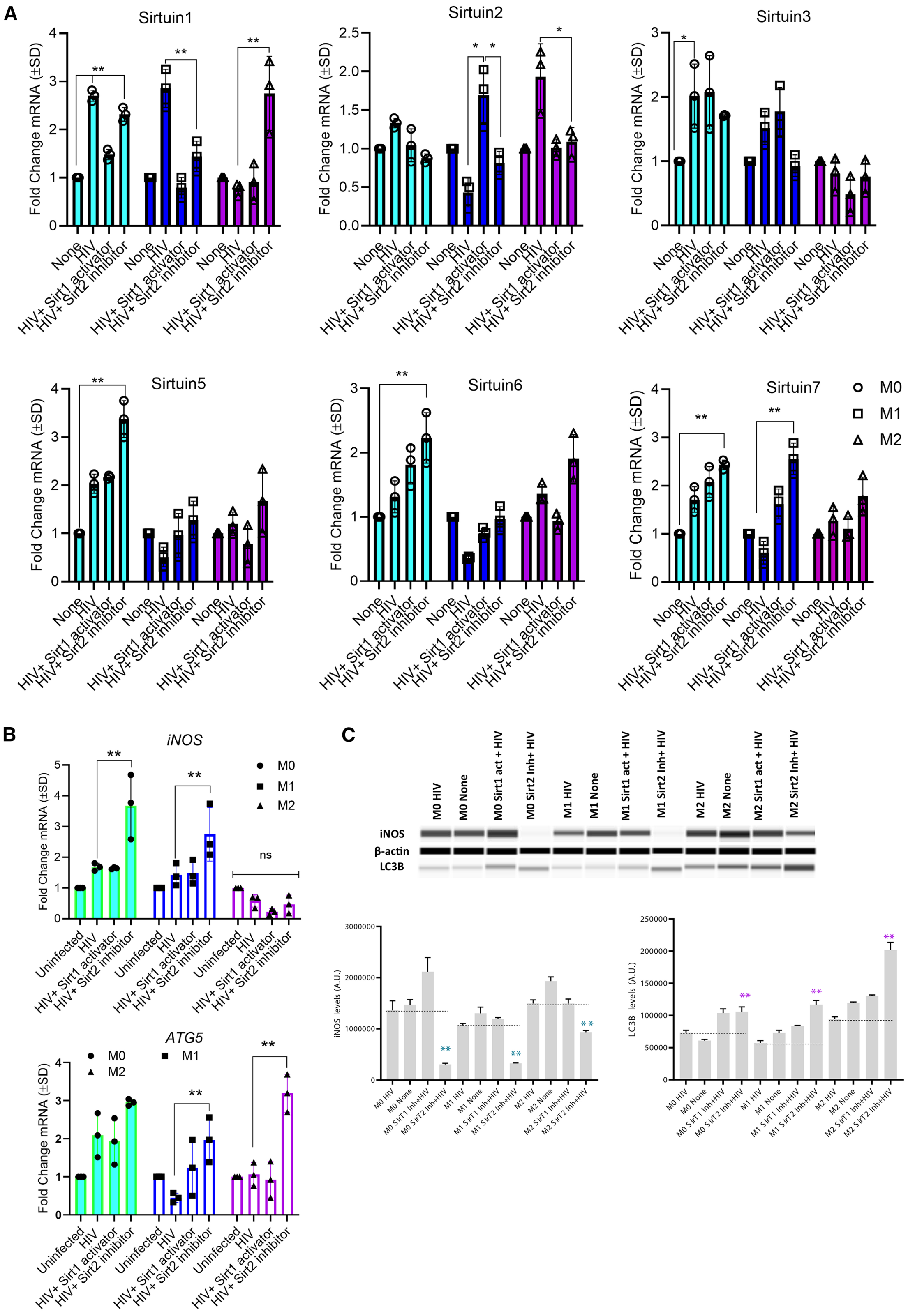

(legend on next page)

alone. Because NF-kB Rel plays both agonistic and antagonistic roles during HIV-1 replication,[17] additional investigations are warranted to define the NF-kB-specific anti-HIV-1 effects of Sirt2 inhibition.

Sirt2 blockade using sirtinol suppresses both HIV and Mtb replication during co-infection of MΦs

Both HIV-1 and Mtb can co-infect MΦs, and our findings indicate that Sirt2 blockade suppresses replication of both pathogens (Figures 1 and 3). Therefore, we investigated whether sirtinol could simultaneously inhibit HIV/Mtb replication in MΦs. Figure 5 shows that M1 and M2-MΦs (rested and subsequently infected with HIV) from four healthy donors exhibited similar levels of HIV-1 RNA loads 3 days post infection, regardless of M1 or M2 phenotype or treatment regimen (sirtinol, cART, or sirtinol/cART). 7 days post-infection, a significant reduction in viral RNA levels was observed in HIV-infected M2-MΦs treated with sirtinol compared with untreated controls. The viral RNA load of the sirtinol-treated M2-MΦs was similar to levels observed following either single cART or combined cART/sirtinol treatment. Co-infection with Mtb did not significantly affect HIV-1 replication, as both M1 and M2-MΦs showed similar viral loads in both the HIV-1-only and HIV/Mtb co-infection groups (Figure 5A). Of note, Mtb bacterial load was also significantly reduced 7 days post infection in HIV/Mtb co-infected M2-MΦs after sirtinol treatment (Figure 5B). Reduced Mtb load was observed only in sirtinol-treated M2-MΦs, with or without additional cART treatment. Conversely, M2-MΦs treated only with cART showed Mtb bacterial loads comparable to those of untreated MΦs, whether infected with Mtb alone or with HIV/Mtb (Figure 5B).

The Hu-NSG-SGM3 mouse model permits replication of both HIV-1 and Mtb infections

To establish an animal model for HIV/Mtb co-infection, we first infected humanized NSG-SGM3 mice with Mtb H37Rv using the aerosol route. In co-infected groups, this was followed by intraperitoneal infection with HIV-1. Mice receiving either Mtb alone or HIV-1 alone and mice left uninfected were used as controls. Post-infection, HIV-1-infected mice exhibited a decreased CD4/CD8 T cell ratio compared with uninfected controls (Figure 6A). Of note, HIV-1 RNA was detected in the humanized mice starting at 28 days post infection (dpi), with a viral RNA load between 10^7 and 10^8 genome copies/mL of serum, with an increase observed by 35 dpi (Figure 6B). Mtb infection was confirmed with detection of a bacillary load of around 100 colony forming units (CFU) in the lungs of control animals euthanized 1 dpi (Figure 6C). At the end of the study (35 dpi), lung Mtb loads ranged from 10^5 to 10^6 CFU in the Mtb single-infection animals and from 10^4 to 10^6 CFU in the HIV/Mtb co-infected mice, though no statistical differences were found between these groups. Pulmonary function tests in these mice demonstrated significantly reduced lung volume and compliance, as well as increased elastance and resistance in the HIV/Mtb co-infected mice compared with uninfected controls (Figures 6D and S1). Computed tomography (CT) scans also showed multiple high-density areas in the lungs of Mtb-infected animals, with and without HIV-1 co-infection (Figure 6E), indicating inflammation and pathological changes. Both lung sections of Mtb single infection and HIV/Mtb co-infection showed typical miliary tuberculosis pathology (Figure S2). Although Mtb was also found in the spleens, the average CFU counts were lower than in the lungs; loads of 1.5×10^4 and 1.8×10^5 in the Mtb-alone and HIV/Mtb co-infection groups, respectively, suggested that HIV-1 co-infection elevated spleen Mtb loads.

Effect of Sirt2 blockade using sirtinol treatment in humanized mice co-infected with HIV/Mtb

To determine the effect of sirtinol on HIV/Mtb co-infection, humanized mice were treated with sirtinol intraperitoneally (Figure 7A). No significant differences in HIV RNA levels were detected in the HIV/Mtb co-infected mice after sirtinol treatment when compared with untreated (but HIV/Mtb co-infected) mice, though one animal exhibited undetectable levels of viral RNA, which had previously been positive by HIV RT-qPCR. However, mice receiving cART alone or cART + sirtinol displayed a significant reduction in viral RNA loads (Figure 7B).

The Mtb bacterial load in the sirtinol-treated mice was significantly lower than that in untreated mice, for both lung and spleen samples (Figures 7C and 7D). No significant differences in Mtb load were found in mice treated with cART alone or those receiving cART + sirtinol therapy. CT scans showed extensive lung lesions in Mtb-infected animals, with or without HIV co-infection, while pulmonary function tests did not show differences between the HIV/Mtb-infected groups with or without sirtinol treatment. This indicated that 18 days of Mtb infection may not be enough to produce significant changes in pulmonary function (Figure S3). Conversely, lungs from sirtinol-treated mice showed smaller lesions that were comparable to those of uninfected mice (Figure 7E).

DISCUSSION

Mtb and HIV-1 cause serious infections that are difficult to control and require complex, multi-drug treatment regimens over extended

Figure 2. Differential expression of sirtuin mRNA affects nitric oxide and autophagy in HIV-1-infected human macrophages
(A) MΦs pooled from 3 donors each, prepared as naive (M0), M1, or M2 as in Figure 1, were infected with HIV-1 (NL-AD8 strain; stock virus concentration of 1×10^6 $TCID_{50}$/mL). For infection, 100 μL of the virus was added to achieve a multiplicity of infection of 0.1 ($TCID_{50}$). Trizol lysates were collected on day 3 post infection. Replicate sets of these samples were treated with a Sirt1 activator (resveratrol, 20 μM) or a Sirt2 inhibitor (sirtinol, 38 μm) and subsequently analyzed using qPCR for the indicated sirtuins (*$p < 0.006$; **$p < 0.007$; two-tailed t test). (B) Trizol lysate-derived mRNA from (*A*) was assayed using primers for human inducible nitric oxide synthase (*iNOS*) and Autophagy regulating gene 5 (*ATG5*). (C) Additional replicate sets of MΦs from the (*B*) were pooled and analyzed for protein expression using mAbs specific for iNOS and light chain associated with microtubule B (LC3B), a biomarker for autophagy. Western blots were conducted using capillary gel electrophoresis (Figure S4). Densitometry quantitation (average units; AU) is shown.

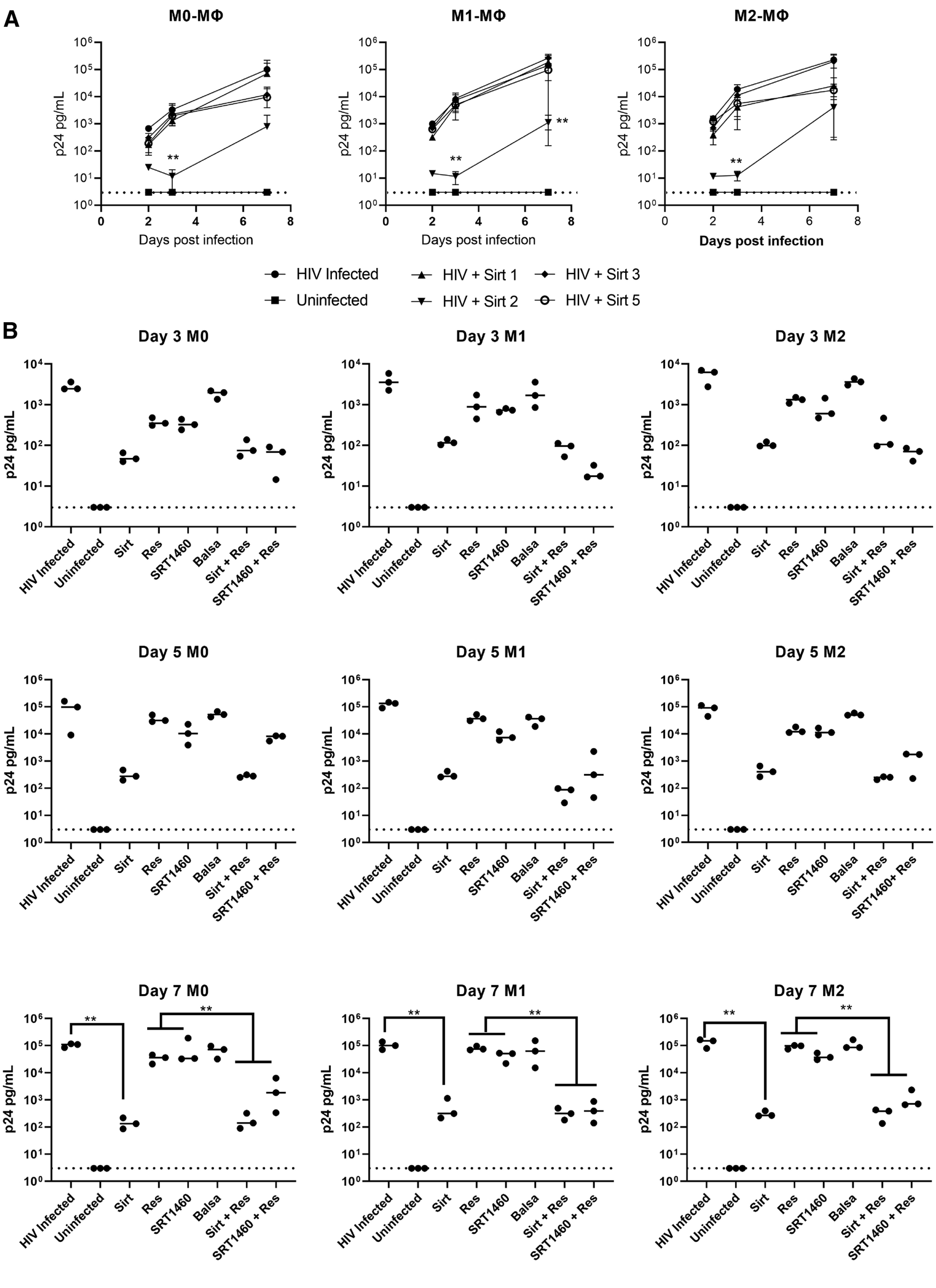

Sirt: Sirtinol (Sirt2 inhibitor) Res: Sirt1 activator Resveratrol
SRT1460: Sirt1 activator Sirt5 inhibitor: Balsalazide

Figure 3. Sirt1 activation and Sirt2 blockade restrict HIV-1 replication in macrophages

(A) Macrophages were infected at an MOI of 0.1 with HIV-1 NLAD8 as described in the materials and methods. After 24 h, infected cells were treated with the indicated Sirt activators or inhibitors. HIV-1 production was quantified by HIV-1 p24 ELISA at 2, 3, and 7 dpi (**$p < 0.007$; two-tailed *t* test). (B) Macrophage infections with HIV-1 NLAD8 were performed as in (*A*), but the drugs were added at 24 h post infection and re-applied on days 3 and 5 post infection followed by HIV-1 p24 ELISA (**$p < 0.01$; two-tailed *t* test).

durations. Both pathogens persist for decades in the human body and can be reactivated from a latent state. While HIV-1 primarily targets CD4 T cells, it can also infect MΦs[19]; recent studies have identified MΦs as a source for the virus in individuals who are unresponsive to cART, thereby rejuvenating research into this relatively unexplored niche.[19] Similarly, Mtb can infect many cell types but

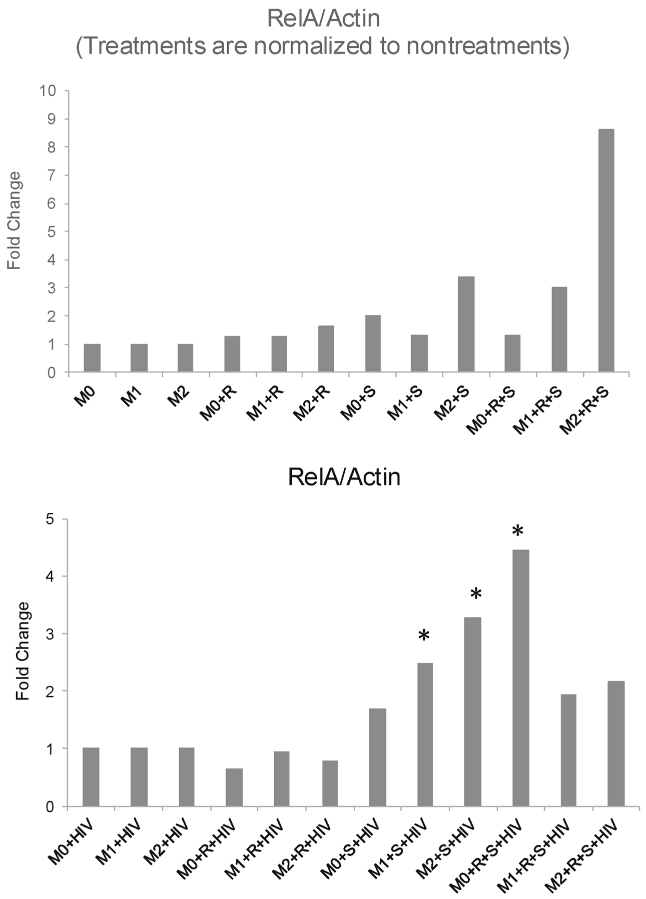

Figure 4. Sirt2 blockade with sirtinol and Sirt1 activation with resveratrol increase Rel-A/NF-kB acetylation

MΦs derived from human donors were either left naive (M0) or differentiated into M1 or M2 phenotypes, followed by HIV-1 infection as described in Figure 3A. Following infection, cells were treated with sirtuin modulators, including sirtinol (38 μM) alone or sirtinol and resveratrol (30 μM). Cell lysates were collected on day 4 after addition of drugs and analyzed for Rel-A acetylation using OMICS. Top: uninfected MΦs. Bottom: HIV-infected M0, M1, and M2-MΦs (**p* < 0.01; vs. HIV alone groups, *t* test). Figure S5 shows raw data analysis.

predominantly replicates and persists within MΦs. For example, Mtb is demonstrable within multi-nucleate giant cells of tuberculosis granulomas, which arise through the fusion of MΦs. Indeed, host-directed therapies for tuberculosis mostly center around the activation of MΦs.[20] Given the emerging importance of MΦs during HIV/Mtb co-infection, we present a novel paradigm that Sirt2 and Sirt1 modulation can control the co-infection.

In our recent study, we found that human MΦs exhibit functional heterogeneity. Specifically, IFN-γ-preconditioned M1-MΦs effectively controlled Mtb growth through autophagy and NO production. In contrast, IL-4-preconditioned M2-MΦs, lacking these antimicrobial mechanisms, permitted increased Mtb growth.[3] These phenotypic differences were reflected in distinct transcriptomic profiles, with M1-MΦs showing stronger Sirt5 expression and M2-MΦs showing marked Sirt2 upregulation.[21] Notably, Sirt2 blockade with sirtinol not only enhanced Mtb clearance in MΦs and C57BL/6 mice (Figure 1), but it also facilitated a phenotypic switch from M2 to M1, as evidenced by dramatic changes in proteomics and antimicrobial function (unpublished findings). Unexpectedly, HIV-1 infection also upregulated Sirt2 in addition to Sirt1 in MΦs (Figure 2). We have recently described that sirtuins play a major role in regulating histone modifications that affect MΦ polarization and antimicrobial function.[16,22] Herein, we demonstrate a novel immunoregulatory role for sirtuins during both standalone and co-infections with Mtb and HIV-1 in human MΦs.

Though the HIV-1-derived Tat protein enhances viral replication by binding to Sirt1 in T cells,[4,5] we found that Sirt2 blockade dramatically inhibits HIV-1 replication in MΦs (Figure 3). Although Sirt1 activation with resveratrol alone did not inhibit HIV-1, its combination with SRT1460 significantly reduced HIV-1 replication in MΦs. Because we used only two doses of these drugs on day 2 and 5 to inhibit HIV-1 *ex vivo,* and because multiple dosing during chemotherapy is feasible, we propose that a longer duration of treatment with multiple or higher drug doses could offer a more effective treatment regimen.

Like Mtb, HIV-1 also induces mRNA transcripts for Sirt1, Sirt2, and Sirt3 and detectable levels of Sirt5, Sirt 6, and Sirt7. It is possible that these latter sirtuins also play a role during the progression of HIV-1 pathogenesis. Sirt2 blockade increased transcripts for Sirt5, Sirt6, and Sirt7 in HIV-1-infected MΦs (Figure 3), suggesting there is a cross-regulatory mechanism among sirtuins that warrants additional investigation. Sirtuins are histone deacetylases that epigenetically modify gene expression. Interestingly, it has been shown that HIV-1 Tat binding to Sirt1 inhibits its activity, thereby increasing acetylation of the NF-kB p50/p65 subunit, which hyperactivates T cells and supports HIV-1 replication.[4,5] This may also explain the increase in the acetylation of Rel-A in HIV-1-infected MΦs (Figure 4). Sirt1 is also critical for Tat-mediated transactivation of HIV-1.[23] These paradoxical observations underscore the dual agonistic and antagonistic functions of NF-kB:Rel and Sirt1 in the context of HIV-1 replication and emphasize the need for additional research.[17]

We also show that sirtinol reduces the replication of HIV-1 in co-infected MΦs and in the organs of humanized mice. The inhibitory effect of sirtinol on HIV-1 was mostly evident during *in vitro* MΦs infections. However, sirtinol alone did not appear to inhibit HIV-1 effectively in mice, as it achieved significant suppression only when combined with cART. Much of the virus quantified in serum originated from $CD4^+$ T cells, potentially masking the inhibitory effect of sirtinol on HIV-1 produced by MΦs in organs, such as the lungs. Further analysis of HIV-1 cell-associated RNA in lung tissue is needed to determine the impact of sirtinol on HIV-1 expression and its potential association with reduction of lesions. Additionally, longer treatment durations with cART and sirtinol may provide additional insights into whether maximal suppression of HIV-1 and Mtb can be accomplished, reducing lesion severity.

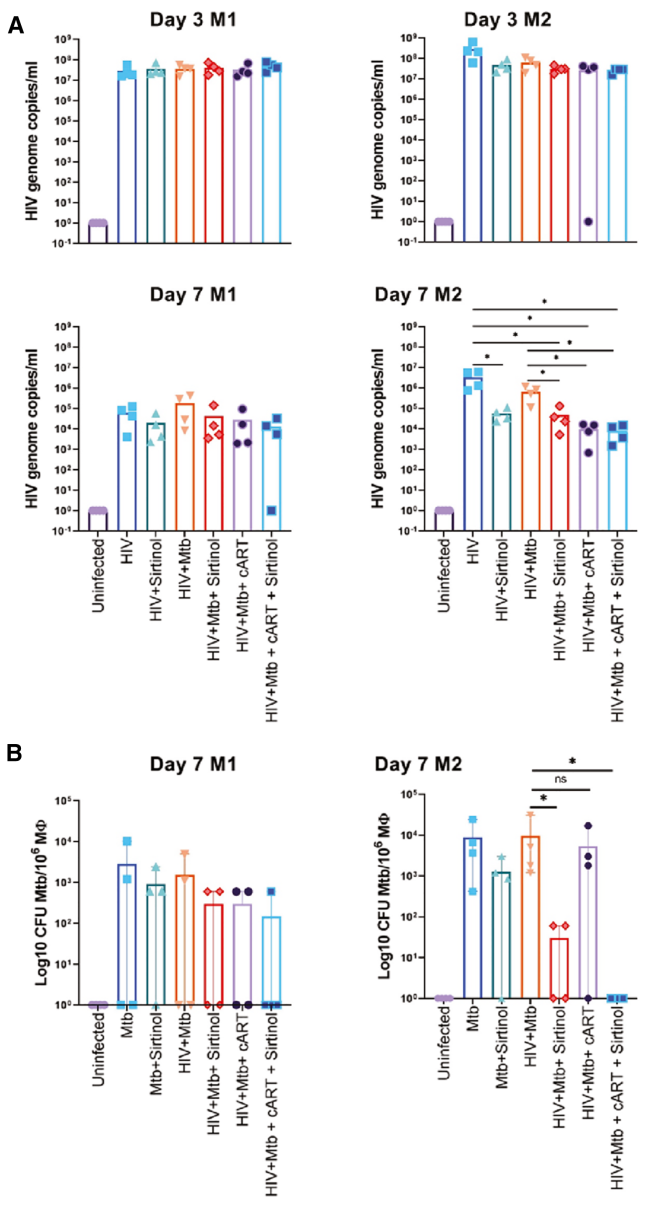

Figure 5. Sirt2 blockade using sirtinol inhibits both HIV and Mtb replication in co-infected macrophages

MΦs from 4 donors were differentiated into M1 and M2 phenotypes before being infected with either HIV, Mtb, or both pathogens together. Then, various treatments were applied as indicated. (A) Viral loads for HIV were assessed from supernatants collected on days 3 and 7; (B) Mtb CFUs were quantified from MΦ lysates on day 7 (*$p < 0.01$; paired t test).

MATERIALS AND METHODS

Macrophages and Mtb strains

Healthy, donor-derived MΦs were purified from PBMCs using CD14 magnetic beads. Their differentiation into M0- (naive), M1-, and M2-MΦs has been described previously.[3] MΦs were first infected using Mtb (MOI = 1) or HIV-1 followed by treatment with sirtuin (1/2/3/5) inhibitors or activators and growth assayed over 7 days using Mtb CFU counts or p24 ELISA. We combined sirtuin modulators with anti-retroviral drugs where indicated. MΦ lysates were assayed for sirtuins, *iNOS*, and *ATG5/7* gene expression, and western blots using Abbie capillary gel electrophoresis were performed as described previously.[3] For all *ex vivo* and C57BL/6 experiments, Mtb strain Erdman was used, whereas we used Mtb H37Rv for humanized mouse experiments. They are nearly identical in growth patterns in these mice.

Cell lines and infectious HIV-1

The R5 macrophage-tropic plasmid proviral clone, pNL(AD8) (NLAD8), and TZM-bl cell line were obtained from the NIH AIDS Reagent Program.[24–26] 293T cells were obtained from the American Type Culture Collection (ATCC, CRL-3216). Both the 293T and TZM-bl cell lines were maintained in complete Dulbecco's modified Eagle's medium (DMEM), i.e., high-glucose (4.5 g/L) (Corning, 10-0130-CV) supplemented with 10% fetal bovine serum heat-inactivated at 56°C for 30 min, 2 mM L-glutamine, 1 mM sodium pyruvate, 100 U/mL penicillin, 100 μg/mL streptomycin. Stocks of infectious HIV-1 NLAD8 were produced by transfection of 293T with pNL(AD8), and the infectious titers were determined by limiting dilution using TZM-bl cells to score infection by luciferase assay, as we have previous described,[27]

Macrophage infection and HIV-1 p24 ELISA

1×10^6 naive, IFN-γ, or IL-4 pre-programmed MΦs in 24-well-plates were infected with HIV-1 NLAD8 strain at an MOI of 0.1 for 24 h. After 24 h, cells were washed once using 1 mL of Dulbecco's phosphate-buffered saline (PBS) without calcium and magnesium (Corning, 21-031-CV) to remove unattached virions. 1 mL of fresh medium was replaced, and supernatants were collected to quantify HIV-1 p24 antigen by antigen capture ELISA (Advanced Bioscience Laboratories, #5447), according to the manufacturer's protocol. Absorbance was determined using a universal micro-plate reader (BIO-TEK instruments ELx800). Test sample HIV-1 p24 concentrations were calculated from a standard curve by linear regression analysis. Significance was determined by the appropriate statistical analysis in GraphPad Prism.

Sirtinol drug assay using C57BL/6J mice

Age- and sex-matched C57BL/6 mice (JAX; n = 5 per group, 4–8 weeks, male and female) were infected with Mtb Erdman (100 CFU per mouse) using a Glas-Col aerosol chamber. On days indicated post infection, mice were injected intraperitoneally with 10 mg/kg body weight sirtinol. Mice were sacrificed on day 30 post infection to assess Mtb CFU counts in the lungs by plating homogenates on 7H10 agar supplemented with oleic acid, albumin, dextrose, and catalase (OADC). Our previously described mouse protocol was used after approval by the Institutional Animal Care and Use Committee (IACUC).[28]

Mtb and HIV co-infection procedures: bacterial and viral strains

Mtb H37Rv was obtained from BEI Resources and propagated in the biosafety level 3 (BSL-3) facilities at the University of Texas Health

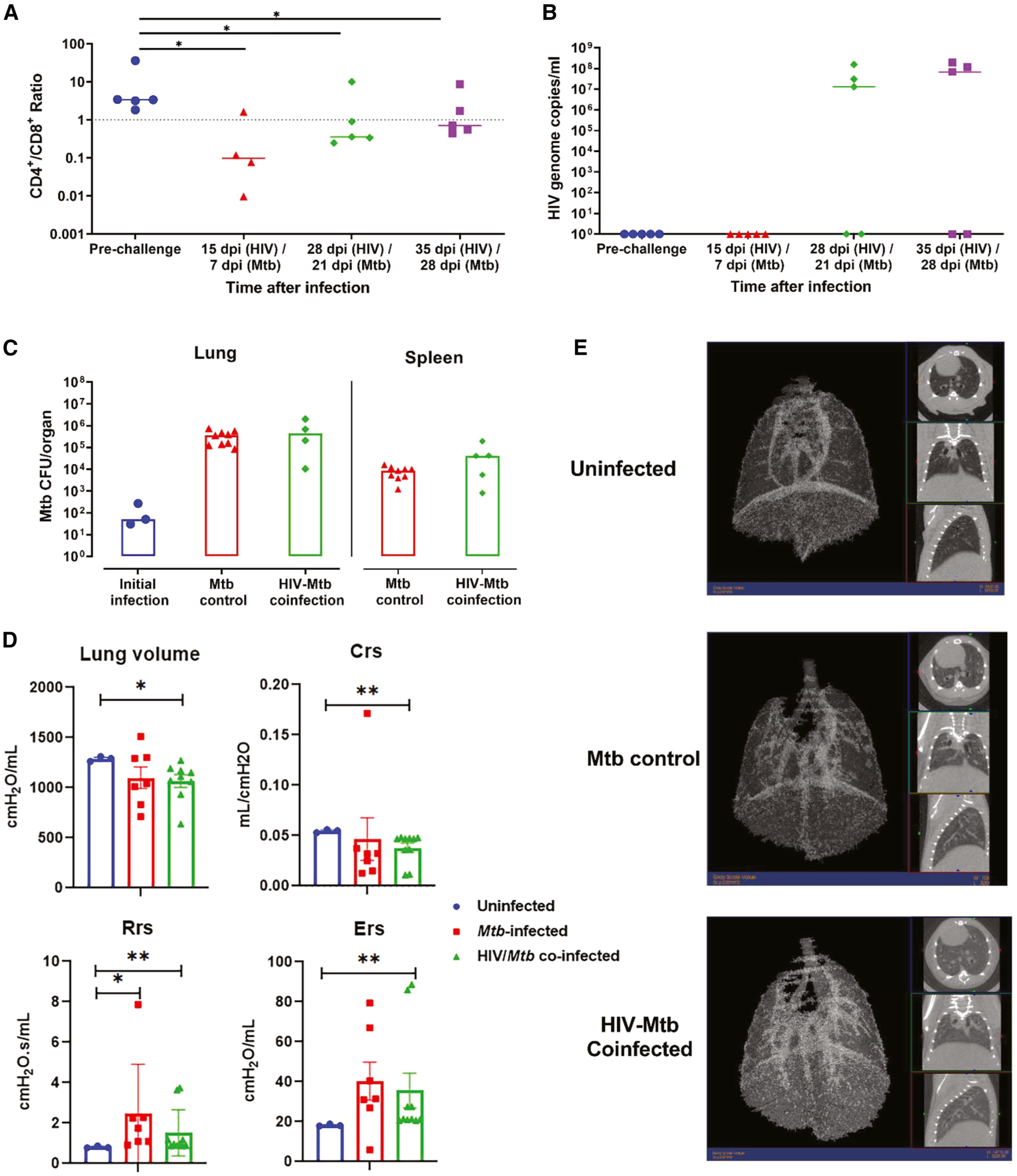

Figure 6. Humanized mice (Hu-NSG-SGM3) allow replication of HIV-1 and Mtb during co-infection

Hu-NSG-SGM3 mice were initially infected intraperitoneally with 10^5 TCID50 of the HIV-1 BAL strain (for groups designated as HIV alone and HIV/Mtb co-infection). 7 days post HIV infection, an aerosolized dose of Mtb H37Rv (100 CFU per mouse lung) was administered using the Madison chamber, as previously described by us[18] in both Mtb-only and HIV/Mtb co-infection groups. Day 0 implantation of Mtb was verified through lung cultures from sacrificed mice. (A and B) CD4+ T cell levels (A) and HIV viral load (B) were monitored across different time points. (C) Mtb bacillary load in lung and spleen tissues was assessed at the study's conclusion. (D) Pulmonary function was evaluated by measuring lung volume and respiratory resistance (Rs), elasticity (Ers), and compliance (Crs). (E) CT scans were performed, and one representative figure is shown for each group. The left panels show the 3D images, with white areas indicating high-density scans (e.g., tissues) and black areas indicating low-density scans (e.g., air). The three smaller figures for each mouse show various scan angles per mouse. Unpaired multiple t tests were used for statistical analysis. (ns: $p \geq 0.05$, no significant difference; *$p < 0.05$; **$p < 0.01$; and ***$p < 0.001$).

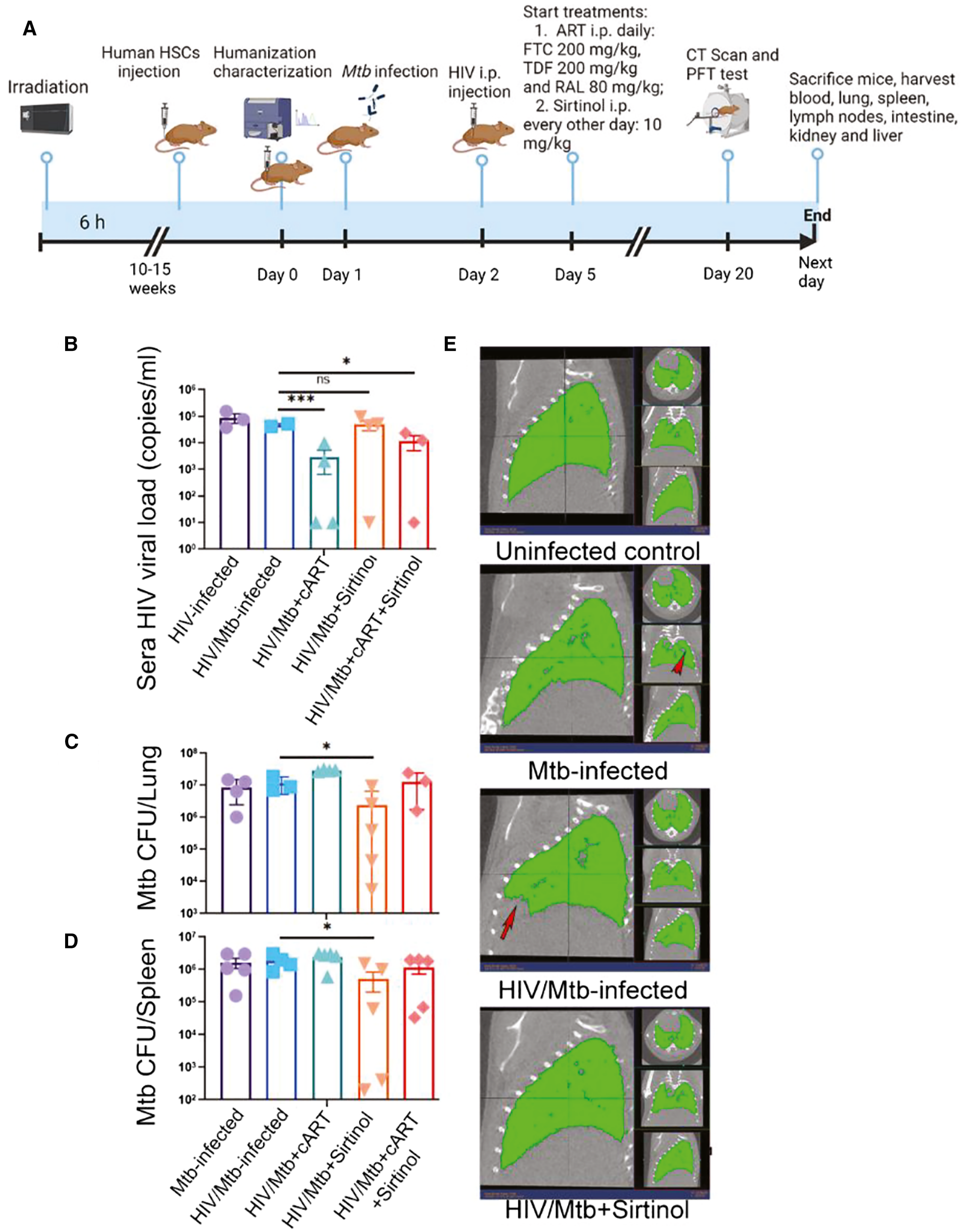

Figure 7. Sirtinol inhibits replication of both HIV/Mtb in Hu-NSG-SGM3 mice

(A) Protocol for co-infection and treatment. Humanized NSG-SGM3 mice were infected with HIV and/or Mtb, followed by administration of various treatments as indicated. cART was administered daily, while sirtinol (10 mg/kg body weight) was given every other day (totaling 12 doses over 24 days). Post treatment, mice were sacrificed for CT scans, followed by collection of sera and tissue samples to determine HIV viral loads and Mtb CFU counts. (B) Serum HIV viral load measurements. (C and D) Mtb CFU counts within the lung (C) and spleen (D). (E) the lung CT scans, with gray areas within the functionally illustrated lungs (green) depicting inflammation or pathological changes (such as lesions, etc.). The obvious lesions visible to the naked eye are marked with red arrows. Unpaired multiple *t* tests were used for statistical analysis (ns: $p \geq 0.05$, no significant difference; *$p < 0.05$; **$p < 0.01$; and ***$p < 0.001$).

Science Center at Tyler (UTHSCT). Mtb was cultured in 7H9 medium with 10% ADC following standard Mtb culture procedures.[29] After 7 days of growth, the bacteria were collected and subjected to sonication three times, at an amplitude of 38%, for 10 s each, with a 5-s interval, followed by low-speed centrifugation (1,100 RPM). Bacteria were diluted to an optical density value of ≈1 in sterile 0.9% NaCl; aliquots were made and frozen at −80°C to be used as inoculum. 2 weeks later, one aliquot was thawed, and the bacterial load was evaluated by plating 10-fold serial dilutions in 7H10 agar, supplemented with OADC. After 2–3 weeks of incubation, the CFUs per aliquot were calculated.

HIV-1 BAL strain

The HIV-1 BAL strain was obtained from the NIH AIDS Reagent Program, and prepared in the BSL-3 facilities at UTHSCT, following standard procedures.[30] Briefly, frozen human PBMCs (STEMCELL Technologies, Vancouver, Canada) were thawed and seeded in a 75-cm^2 flask at a concentration of 5 × 10^6 cells/mL in RPMI-1640 medium (Corning Inc., Corning, NY) supplemented with 10% fetal bovine serum (FBS), 1% penicillin/streptomycin, 1 μg/mL of PHA, and 2 μg/mL polybrene (Millipore Sigma, Burlington, MA). After 3 days of stimulation, 4 × 10^7 cells were centrifuged and infected with 1.5 mL of HIV-1 in two adsorption cycles. Following the second adsorption cycle, the cells were seeded in two 75-cm^2 flasks with 30 mL of medium supplemented with FBS, antibiotics, and human IL-2 (20 units/mL). Cell culture supernatant was collected every 3 days, with fresh medium being added, until day 21 of culture and stored at −80°C.

Animal experiments using humanized mice

All animal procedures were approved by the UTHSCT IACUC. NOD.Cg-Prkdcscid Il2rgtm1Wjl Tg(CMV-IL3,CSF2,KITLG)1Eav/MLoySzJ (NSG-SGM3) mice were purchased from The Jackson Laboratory (Bar Harbor, ME) and bred in the vivarium facilities at UTHSCT. Pups were weaned at 21 days after birth, and 1–3 weeks after that, they were irradiated at a dose of 100 cGy/mouse, followed by intravenous injection with 2 × 10^5 CD34$^+$ stem cells/mouse at 12 h post irradiation. Humanization was monitored starting at 12 weeks after stem cell transplantation and again at 14 and 16 weeks. For this purpose, blood was drawn from the submandibular vein (100–150 μL, based on animal weight), and PBMCs were collected through density gradient centrifugation using Ficoll-Paque (Cytiva, Marlborough, MA). After erythrocyte lysis, PBMCs from each animal were stained for the human (hu) and mouse (mo) hematopoietic cell surface marker CD45$^+$, as well as lymphocytic and myeloid markers. Animals that showed a positive huCD45$^+$/moCD45$^+$ ratio, accompanied by differentiation of various immune cell populations, were selected for experimental infection.

Mice were randomly divided into four experimental groups: uninfected (n = 5), HIV-infected (n = 8), Mtb-infected (n = 8) and HIV/Mtb co-infected (n = 7). Mtb infection was performed using aerosolized Mtb H37Rv through a Madison chamber, as previously described,[31] using an infection dose of 100 CFU/lung. Three additional mice were included in the Madison chamber at the time of infection and were euthanized 24 h after infection. The lung sample was collected, macerated, and cultured in 7H10 agar to confirm the initial bacterial load of infection.[18]

1 day after Mtb infection, the mice from the single HIV infection and HIV/Mtb co-infection groups were subjected to intraperitoneal inoculation with 10^5 TCID50 of HIV-1 BAL strain. Blood samples from all experimental groups were collected on the day of infection and at 15, 28, and 35 dpi. Serum samples from all animals were separated and stored at −80°C until further use. PBMCs were isolated and stained for flow cytometry analysis. At 35 dpi, animals were terminally anesthetized using a ketamine/xylazine mixture to perform CT scans and pulmonary function (PF) tests. Afterward, the animals were euthanized, and whole-blood samples were collected through cardiac punction. During necropsy, lung and spleen samples were collected and macerated through a 70-μM cell strainer (Thermo Scientific) to a final volume of 2 mL of PBS. Serial 10-fold dilutions of the organ macerates were plated in 7H10 agar and supplemented with OADC to assess bacterial load. The remaining volumes of lung and spleen macerates were stored at −80°C for further analysis.

For each experimental group, a lung sample from a single animal was selected for histopathological analysis and, therefore, not subjected to maceration and bacterial culture. Lungs were filled with 10% formalin before being removed from the animal and stored in the same medium after the necropsy.[32,33] Sample processing and hematoxylin and eosin staining was carried out at the histopathology core of UT southwestern.

CT scan and PF testing

The humanized mice were subjected to CT scans and pulmonary function tests before sacrifice. Mice were intraperitoneally injected with ketamine/xylazine (100 mg/kg ketamine, 20 mg/kg xylazine). Once the correct anesthetic plane was achieved, mice were intubated with a sterile, 20G intravenous cannula through the vocal cords into the trachea. Following intubation, anesthesia was maintained using isoflurane. Elastance (Ers), compliance (Crs), and total lung resistance (Rrs) was assessed for each mouse through the snapshot perturbation method, as previously described.[18] Measurements were performed in triplicate for each animal using the FlexiVent system (SCIREQ, Tempe, AZ), with a tidal volume of 30 mL/kg at a frequency of 150 breaths/min against 2–3 cm H_2O-positive end-expiratory pressure. After PF testing, mice were subjected to CT scans to measure lung volume using the Explore Locus Micro-CT Scanner (General Electric, GE Healthcare, Wauwatosa, WI). CT scans were performed during full inspiration and at a resolution of 93 μm. Lung volumes were calculated from lung renditions collected at full inspiration. Microview software 2.2 (http://microview.sourceforge.net) was used to analyze lung volumes and render three-dimensional (3D) images. For CT scan data presentation, we employed 3D lung and green-filled lung pictures. In 3D lung figures, the low-density areas (darker areas) represent well-aerated alveoli,

while the high-density regions (brighter areas) indicate blood vessels and tissues. In tuberculosis disease, if infected mice have much higher density areas than healthy mice, it usually indicates inflammation and other pathological changes. In green-filled figures, the green-filled areas represent the air-filled spaces of the lung, while the dark color areas indicate either flattened tissues or pathological regions.

RNA extraction and RT-qPCR

Serum samples from all experimental groups were extracted using the NucleoSpin RNA isolation kit (Macherey-Nagel, Allentown, PA). Following viral RNA extraction, samples were evaluated using RT-qPCR to determine the viral RNA load in each animal. Control standards (obtained from NIH AIDS Reagent Program) with known quantities of HIV-1 genome copies were used as amplification controls, as well as to establish a standard curve that was used to determine the viral RNA load based on the cycle threshold (Ct) value.

DATA AND CODE AVAILABILITY

All data are contained in this manuscript and the supplemental information.

ACKNOWLEDGMENTS

This work was supported in part by the Texas Developmental Center for AIDS Research, an NIH funded program (P30 AI-161943), NIH AI-116167 (J.T.K), NIH AI-161015 (C.J., J.J.E.), NIH AI-184551 (C.J., J.J.E., G.Y.), and NIH AI-150550 (G.Y.). F.B. was supported by Deutsche Forschungsgemeinschaft with funds from SFB1309 (Chemical Biology of Epigenetic Modifications), Project-ID: 325871075–SFB1309. The authors are grateful to Dr. Heather McConnell for editing the manuscript.

AUTHOR CONTRIBUTIONS

Authors performed experiments as follows: A.M. (Figures 1 and 2), V.K.S., K.T., S.S., A.K. (Figure 3), and K.Z. (Figure 4); F.B. provided reagents; J.A.B and G.Y. performed humanized mice (Figures 5, 6, and 7); and J.J.E., M.E., A.P.R., J.T.K., G.Y., and C.J. provided experimental design, data analysis, and manuscript writing.

DECLARATION OF INTERESTS

The authors declare no conflict of interest and certify that all studies were conducted under approved institutional hematopoietic stem cell and Institutional Review Board protocols.

SUPPLEMENTAL INFORMATION

Supplemental information can be found online at https://doi.org/10.1016/j.ymthe.2026.03.019.

REFERENCES

1. Kruize, Z., and Kootstra, N.A. (2019). The Role of Macrophages in HIV-1 Persistence and Pathogenesis. Front. Microbiol. *10*, 2828. https://doi.org/10.3389/fmicb.2019.02828.

2. Castellano, P., Prevedel, L., Valdebenito, S., and Eugenin, E.A. (2019). HIV infection and latency induce a unique metabolic signature in human macrophages. Sci. Rep. *9*, 3941. https://doi.org/10.1038/s41598-019-39898-5.

3. Khan, A., Zhang, K., Singh, V.K., Mishra, A., Kachroo, P., Bing, T., Won, J.H., Mani, A., Papanna, R., Mann, L.K., et al. (2022). Human M1 macrophages express unique innate immune response genes after mycobacterial infection to defend against tuberculosis. Commun. Biol. *5*, 480. https://doi.org/10.1038/s42003-022-03387-9.

4. Kwon, H.S., Brent, M.M., Getachew, R., Jayakumar, P., Chen, L.F., Schnolzer, M., McBurney, M.W., Marmorstein, R., Greene, W.C., and Ott, M. (2008). Human immunodeficiency virus type 1 Tat protein inhibits the SIRT1 deacetylase and induces T cell hyperactivation. Cell Host Microbe *3*, 158–167. https://doi.org/10.1016/j.chom.2008.02.002.

5. Pinzone, M.R., Cacopardo, B., Condorelli, F., Di Rosa, M., and Nunnari, G. (2013). Sirtuin-1 and HIV-1: an overview. Curr. Drug Targets *14*, 648–652. https://doi.org/10.2174/1389450111314060005.

6. Cheng, C.Y., Gutierrez, N.M., Marzuki, M.B., Lu, X., Foreman, T.W., Paleja, B., Lee, B., Balachander, A., Chen, J., Tsenova, L., et al. (2017). Host sirtuin 1 regulates mycobacterial immunopathogenesis and represents a therapeutic target against tuberculosis. Sci. Immunol. *2*, eaaj1789. https://doi.org/10.1126/sciimmunol.aaj1789.

7. Yang, H., Chen, J., Chen, Y., Jiang, Y., Ge, B., and Hong, L. (2021). Sirt1 activation negatively regulates overt apoptosis in Mtb-infected macrophage through Bax. Int. Immunopharmacol. *91*, 107283. https://doi.org/10.1016/j.intimp.2020.107283.

8. Wu, Q.J., Zhang, T.N., Chen, H.H., Yu, X.F., Lv, J.L., Liu, Y.Y., Liu, Y.S., Zheng, G., Zhao, J.Q., Wei, Y.F., et al. (2022). The sirtuin family in health and disease. Signal Transduct. Target. Ther. *7*, 402. https://doi.org/10.1038/s41392-022-01257-8.

9. Yang, H., Hu, J., Chen, Y.J., and Ge, B. (2019). Role of Sirt1 in innate immune mechanisms against Mycobacterium tuberculosis via the inhibition of TAK1 activation. Arch. Biochem. Biophys. *667*, 49–58. https://doi.org/10.1016/j.abb.2019.04.006.

10. Morselli, E., Maiuri, M.C., Markaki, M., Megalou, E., Pasparaki, A., Palikaras, K., Criollo, A., Galluzzi, L., Malik, S.A., Vitale, I., et al. (2010). The life span-prolonging effect of sirtuin-1 is mediated by autophagy. Autophagy *6*, 186–188. https://doi.org/10.4161/auto.6.1.10817.

11. Zhang, S., Liu, Y., Zhou, X., Ou, M., Xiao, G., Li, F., Wang, Z., Wang, Z., Liu, L., and Zhang, G. (2021). Sirtuin 7 Regulates Nitric Oxide Production and Apoptosis to Promote Mycobacterial Clearance in Macrophages. Front. Immunol. *12*, 779235. https://doi.org/10.3389/fimmu.2021.779235.

12. Liu, X., Jana, M., Dasgupta, S., Koka, S., He, J., Wood, C., and Pahan, K. (2002). Human immunodeficiency virus type 1 (HIV-1) tat induces nitric-oxide synthase in human astroglia. J. Biol. Chem. *277*, 39312–39319. https://doi.org/10.1074/jbc.M205107200.

13. Cairoli, E., Scott-Algara, D., Pritsch, O., Dighiero, G., and Cayota, A. (2008). HIV-1 induced decrease of nitric oxide production and inducible nitric oxide synthase expression during in vivo and in vitro infection. Clin. Immunol. *127*, 26–33. https://doi.org/10.1016/j.clim.2007.11.015.

14. Daskalaki, M.G., Tsatsanis, C., and Kampranis, S.C. (2018). Histone methylation and acetylation in macrophages as a mechanism for regulation of inflammatory responses. J. Cell. Physiol. *233*, 6495–6507. https://doi.org/10.1002/jcp.26497.

15. Zaikos, T.D., Painter, M.M., Sebastian Kettinger, N.T., Terry, V.H., and Collins, K.L. (2018). Class 1-Selective Histone Deacetylase (HDAC) Inhibitors Enhance HIV Latency Reversal while Preserving the Activity of HDAC Isoforms Necessary for Maximal HIV Gene Expression. J. Virol. *92*, 10. https://doi.org/10.1128/JVI.02110-17.

16. Sowers, M.L., Tang, H., Singh, V.K., Khan, A., Mishra, A., Restrepo, B.I., Jagannath, C., and Zhang, K. (2022). Multi-OMICs analysis reveals metabolic and epigenetic changes associated with macrophage polarization. J. Biol. Chem. *298*, 102418. https://doi.org/10.1016/j.jbc.2022.102418.

17. Chan, J.K.L., and Greene, W.C. (2011). NF-kappaB/Rel: agonist and antagonist roles in HIV-1 latency. Curr. Opin. HIV AIDS *6*, 12–18. https://doi.org/10.1097/COH.0b013e32834124fd.

18. Yang, F., Labani-Motlagh, A., Bohorquez, J.A., Moreira, J.D., Ansari, D., Patel, S., Spagnolo, F., Florence, J., Vankayalapati, A., Sakai, T., et al. (2024). Bacteriophage therapy for the treatment of Mycobacterium tuberculosis infections in humanized mice. Commun. Biol. *7*, 294. https://doi.org/10.1038/s42003-024-06006-x.

19. Wong, M.E., Jaworowski, A., and Hearps, A.C. (2019). Corrigendum: The HIV Reservoir in Monocytes and Macrophages. Front. Immunol. *10*, 2517. https://doi.org/10.3389/fimmu.2019.02517.

20. Wallis, R.S., and Hafner, R. (2015). Advancing host-directed therapy for tuberculosis. Nat. Rev. Immunol. *15*, 255–263. https://doi.org/10.1038/nri3813.

21. Zhang, K., Mishra, A., and Jagannath, C. (2024). New insight into arginine and tryptophan metabolism in macrophage activation during tuberculosis. Front. Immunol. *15*, 1363938. https://doi.org/10.3389/fimmu.2024.1363938.

22. Zhang, K., Sowers, M.L., Cherryhomes, E.I., Singh, V.K., Mishra, A., Restrepo, B.I., Khan, A., and Jagannath, C. (2023). Sirtuin-dependent metabolic and epigenetic regulation of macrophages during tuberculosis. Front. Immunol. *14*, 1121495. https://doi.org/10.3389/fimmu.2023.1121495.

23. Zhang, H.S., and Wu, M.R. (2009). SIRT1 regulates Tat-induced HIV-1 transactivation through activating AMP-activated protein kinase. Virus Res. *146*, 51–57. https://doi.org/10.1016/j.virusres.2009.08.005.

24. Freed, E.O., Englund, G., and Martin, M.A. (1995). Role of the basic domain of human immunodeficiency virus type 1 matrix in macrophage infection. J. Virol. *69*, 3949–3954. https://doi.org/10.1128/JVI.69.6.3949-3954.1995.

25. Derdeyn, C.A., Decker, J.M., Sfakianos, J.N., Wu, X., O'Brien, W.A., Ratner, L., Kappes, J.C., Shaw, G.M., and Hunter, E. (2000). Sensitivity of human immunodeficiency virus type 1 to the fusion inhibitor T-20 is modulated by coreceptor specificity defined by the V3 loop of gp120. J. Virol. *74*, 8358–8367. https://doi.org/10.1128/jvi.74.18.8358-8367.2000.

26. Wei, X., Decker, J.M., Liu, H., Zhang, Z., Arani, R.B., Kilby, J.M., Saag, M.S., Wu, X., Shaw, G.M., and Kappes, J.C. (2002). Emergence of resistant human immunodeficiency virus type 1 in patients receiving fusion inhibitor (T-20) monotherapy. Antimicrob. Agents Chemother. *46*, 1896–1905. https://doi.org/10.1128/AAC.46.6.1896-1905.2002.

27. Misra, A., Gleeson, E., Wang, W., Ye, C., Zhou, P., and Kimata, J.T. (2018). Glycosyl-Phosphatidylinositol-Anchored Anti-HIV Env Single-Chain Variable Fragments Interfere with HIV-1 Env Processing and Viral Infectivity. J. Virol. *92*, 10. https://doi.org/10.1128/JVI.02080-17.

28. Khan, A., Bakhru, P., Saikolappan, S., Das, K., Soudani, E., Singh, C.R., Estrella, J.L., Zhang, D., Pasare, C., Ma, Y., et al. (2019). An autophagy-inducing and TLR-2 activating BCG vaccine induces a robust protection against tuberculosis in mice. NPJ Vaccin. *4*, 34. https://doi.org/10.1038/s41541-019-0122-8.

29. Wang, X., Barnes, P.F., Huang, F., Alvarez, I.B., Neuenschwander, P.F., Sherman, D.R., and Samten, B. (2012). Early secreted antigenic target of 6-kDa protein of Mycobacterium tuberculosis primes dendritic cells to stimulate Th17 and inhibit Th1 immune responses. J. Immunol. *189*, 3092–3103. https://doi.org/10.4049/jimmunol.1200573.

30. van 't Wout, A.B., Schuitemaker, H., and Kootstra, N.A. (2008). Isolation and propagation of HIV-1 on peripheral blood mononuclear cells. Nat. Protoc. *3*, 363–370. https://doi.org/10.1038/nprot.2008.3.

31. Feng, Y., Kong, Y., Barnes, P.F., Huang, F.F., Klucar, P., Wang, X., Samten, B., Sengupta, M., Machona, B., Donis, R., et al. (2011). Exposure to cigarette smoke inhibits the pulmonary T-cell response to influenza virus and Mycobacterium tuberculosis. Infect. Immun. *79*, 229–237. https://doi.org/10.1128/IAI.00709-10.

32. Morton, J., and Snider, T.A. (2017). Guidelines for collection and processing of lungs from aged mice for histological studies. Pathobiol. Aging Age Relat. Dis. *7*, 1313676. https://doi.org/10.1080/20010001.2017.1313676.

33. Bohórquez, J.A., Adduri, S., Ansari, D., John, S., Florence, J., Adejare, O., Singh, G., Konduru, N.V., Jagannath, C., and Yi, G. (2024). A novel humanized mouse model for HIV and tuberculosis co-infection studies. Front. Immunol. *15*, 1395018. https://doi.org/10.3389/fimmu.2024.1395018.

YMTHE, Volume *34*

Supplemental Information

Sirtuin2 blockade inhibits replication of human immunodeficiency virus-1 and *Mycobacterium tuberculosis* in macrophages and humanized mice

Vipul K. Singh, Abhishek Mishra, Khanghy Truong, Jose Alejandro Bohorquez, Suman Sharma, Arshad Khan, Franz Bracher, Kangling Zhang, Janice J. Endsley, Mark Endsley, Andrew P. Rice, Jason T. Kimata, Guohua Yi, and Chinnaswamy Jagannath

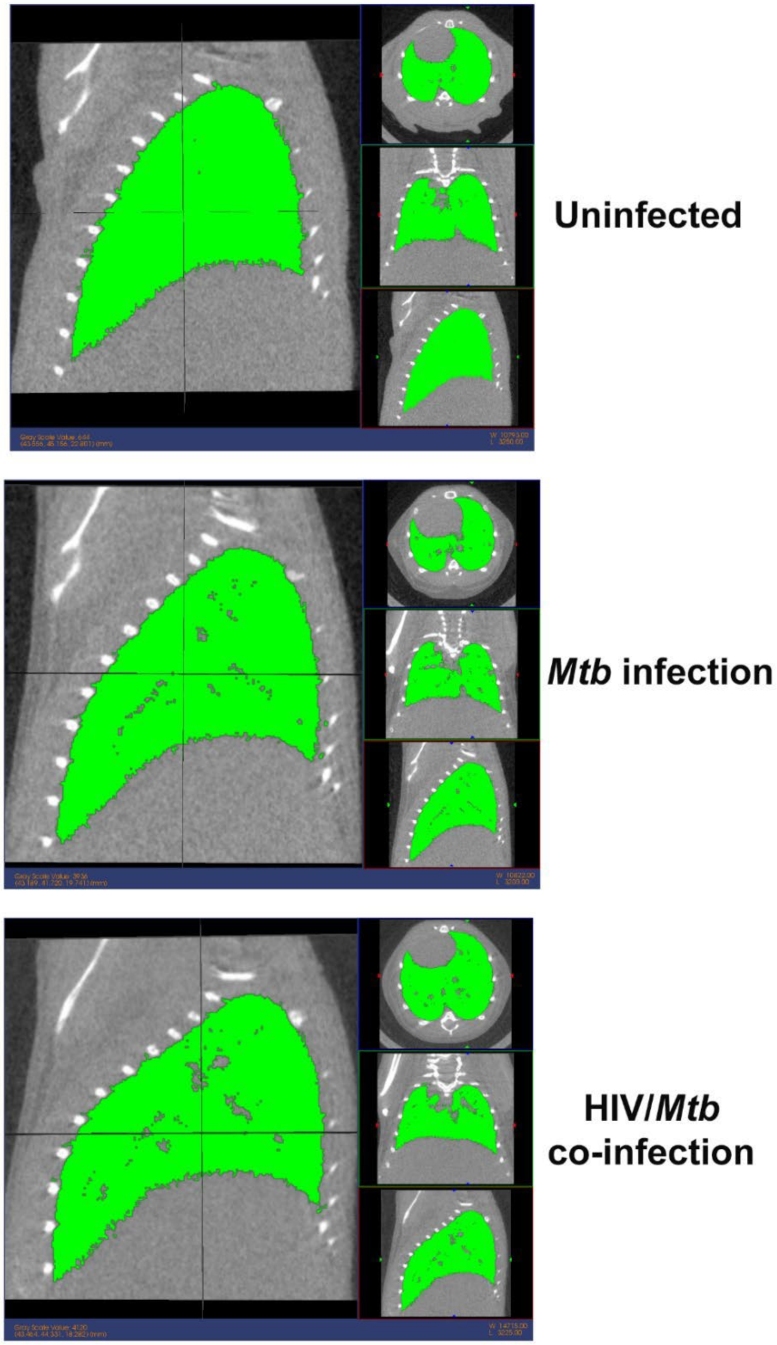

Figure S1: Representative lung volume pictures (green-filled) from animals in different experimental groups.

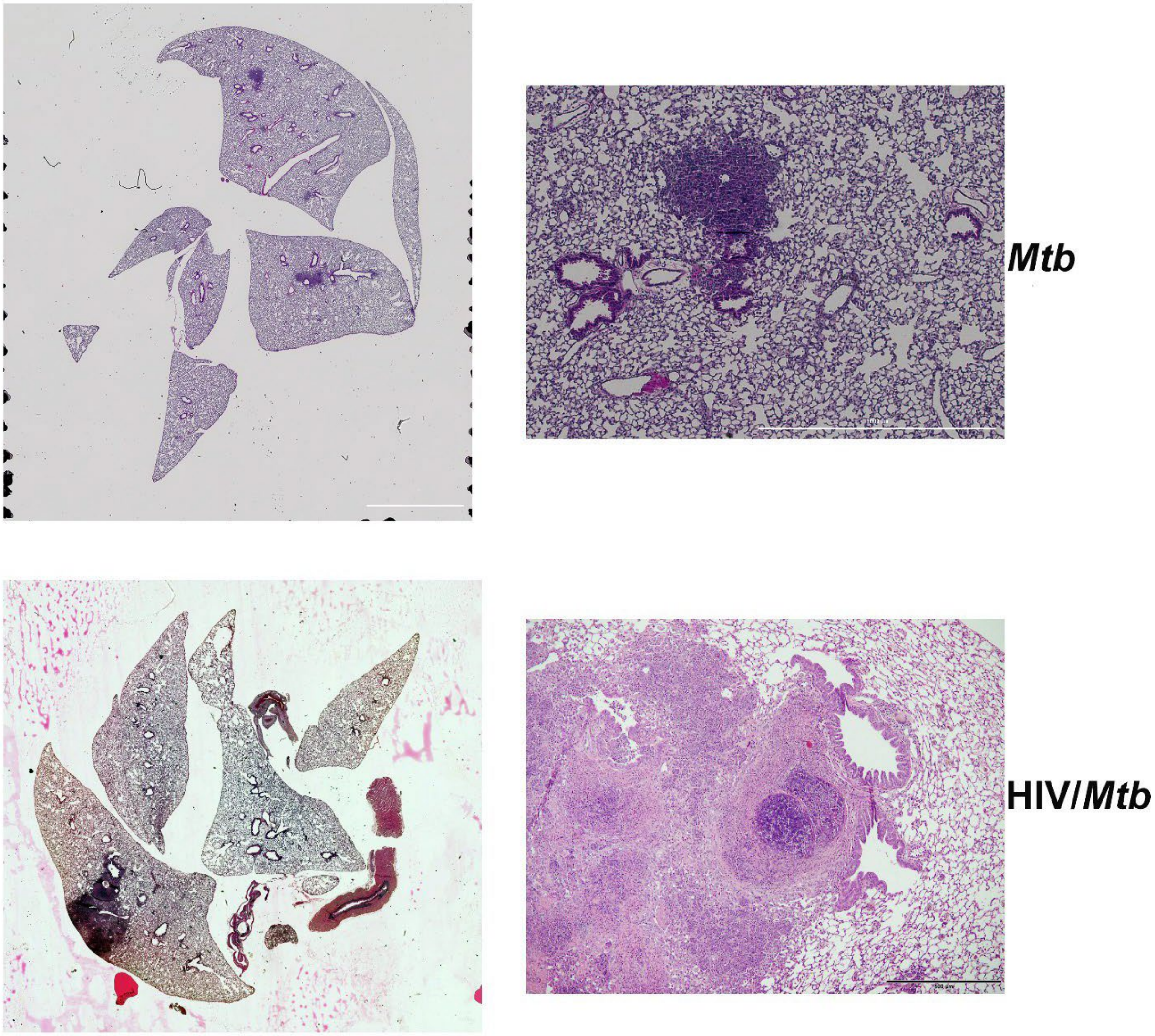

Figure S2: H&E staining of lung sections from representative *M.tuberculosis (Mtb)*-infected mouse and HIV/Mtb co-infected mouse show typical miliary TB pathology, caused by granuloma formation. The left panel shows the whole lungs, and the right panel shows typical granuloma structures in each group.

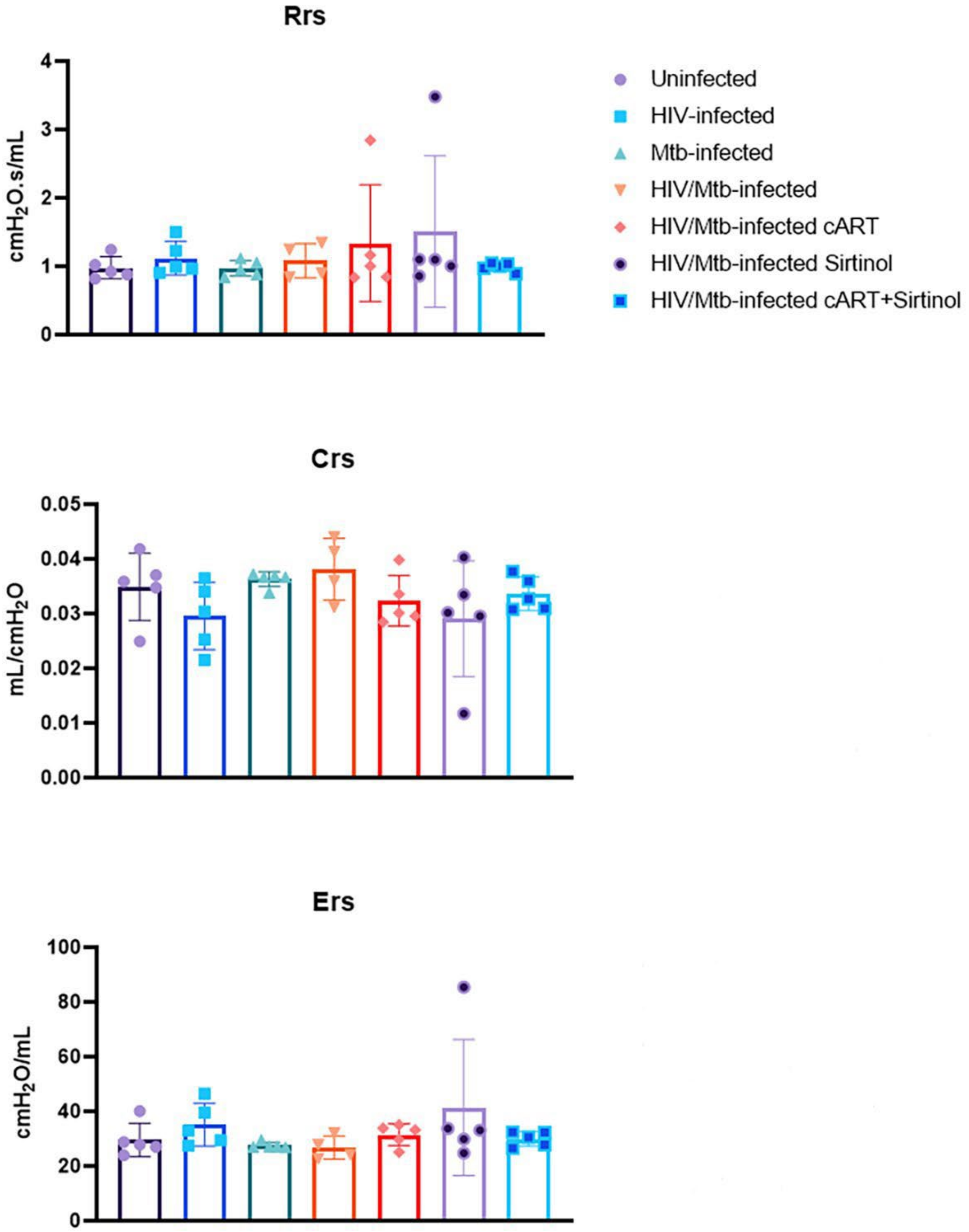

Figure S3: Pulmonary function test parameters: Resistance (Rrs), compliance (Crs) and elastance (Ers), were collected from animals in different treatment groups at the end of the experiment.

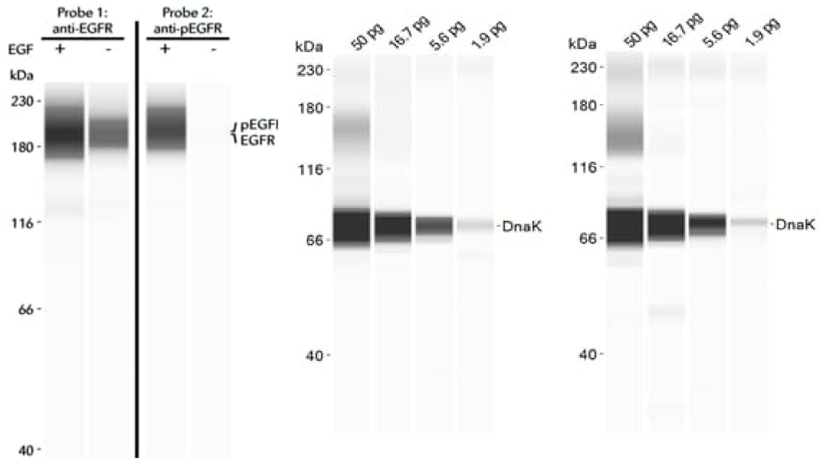

Figure S4: (Figure 2C) Illustration of capillary gel electrophoresis (samples are in individual lanes with markers; gels do not involve cutting and reproducing blots)

Abby's fully analyzed results can be viewed in either lane or electropherogram views. You can use the lane view option to compare band intensity or quantitatively analyze protein size and concentration. Compare the expression of proteins of interest to total protein. Data analysis with Compass's user-friendly software is quick and easy. Save graph and baseline options with run files and quickly annotate lane view results with Compass lane view features. *Lane annotation with Compass 6.0* saves hours spent annotating data in other programs. Abby is a trademark of ProteinSimple, Corp.(blots are reproduced from Abby website.

Name	Signal	Actin	Area	Bkgnd.	Type	Image Na	Channel	Name	Signal	ReA1	Area	Bkgnd.	Type	Ratio	Ratio	Average	STDV	Name	Average		
1	31	35.3	1410	0.00306	Signal	0001692_	800	25	0.0323	0.103	1334	5.29E-05	Signal	0.00292	0.0023	0.0026	0.0004	M0	0.00263	1	
2	39.8	44	1628	0.00257	Signal	0001692_	800	26	0.0386	0.105	1271	5.23E-05	Signal	0.00239	0.0022	0.0023	0.0001	M1	0.00232	1	
3	54.1	58.1	1462	0.00276	Signal	0001692_	800	27	0.0553	0.115	1131	5.26E-05	Signal	0.00198	0.0017	0.0019	0.0002	M2	0.00186	1	
4	18.1	22.1	1088	0.00373	Signal	0001692_	800	28	0.0297	0.0755	864	0.000053	Signal	0.00342	0.0033	0.0033	0.0001	M0+R	0.00334	1.26939	
5	27.3	31.4	1400	0.00291	Signal	0001692_	800	29	0.037	0.0929	1064	5.25E-05	Signal	0.00296	0.0029	0.0029	0.0000	M1+R	0.00292	1.26175	
6	25.2	29.4	1160	0.00361	Signal	0001692_	800	30	0.0357	0.0962	1200	5.04E-05	Signal	0.00327	0.0028	0.0030	0.0004	M2+R	0.00302	1.619351	
7	5.03	7.03	650	0.00308	Signal	0001692_	800	31	0.00479	0.035	616	4.91E-05	Signal	0.00498	0.0057	0.0053	0.0005	M0+S	0.00533	2.024864	
8	24.7	28.1	1073	0.00318	Signal	0001692_	800	32	0.0354	0.0881	1064	4.95E-05	Signal	0.00314	0.0028	0.0030	0.0002	M1+S	0.00299	1.291252	
9	11	14.6	1170	0.00305	Signal	0001692_	800	33	0.0398	0.0943	1073	5.08E-05	Signal	0.00646	0.0062	0.0063	0.0002	M2+S	0.00631	3.386049	
10	23.7	27.5	1295	0.00299	Signal	0001692_	800	34	0.0456	0.0995	1064	5.07E-05	Signal	0.00362	0.0034	0.0035	0.0002	M0+R+S	0.00349	1.324758	
11	4.84	7.14	900	0.00256	Signal	0001692_	800	35	0.00462	0.0438	825	4.75E-05	Signal	0.00613	0.0079	0.0070	0.0012	M1+R+S	0.00699	3.01784	
12	0.615	3.62	1326	0.00227	Signal	0001692_	800	36	0.00323	0.0448	880	4.73E-05	Signal	0.01238	0.0197	0.0160	0.0052	M2+R+S	0.01602	8.604058	
13	34.4	38.5	1440	0.00282	Signal	0001692_	800	37	0.042	0.129	1680	5.18E-05	Signal	0.00335	0.0028	0.0031	0.0004	M0+HIV	0.00307	1.166172	1
14	42	46	1496	0.00268	Signal	0001692_	800	38	0.0523	0.126	1386	5.28E-05	Signal	0.00274	0.0029	0.0028	0.0001	M1+HIV	0.00280	1.06568	1
15	41.2	45.1	1440	0.00269	Signal	0001692_	800	39	0.0605	0.131	1320	5.36E-05	Signal	0.00290	0.0029	0.0029	0.0000	M2+HIV	0.00293	1.571681	1
16	58	62.1	1386	0.00301	Signal	0001692_	800	40	0.0459	0.12	1320	5.64E-05	Signal	0.00193	0.0020	0.0020	0.0000	M0+R+HIV	0.00197		0.640758
17	43.5	47.4	1365	0.00282	Signal	0001692_	800	41	0.0507	0.113	1147	5.41E-05	Signal	0.00238	0.0028	0.0026	0.0003	M1+R+HIV	0.00261		0.931704
18	60.3	64.6	1505	0.00286	Signal	0001692_	800	42	0.0727	0.139	1240	5.36E-05	Signal	0.00215	0.0025	0.0023	0.0002	M2+R+HIV	0.00232		0.792019
19	18.5	21.6	1188	0.00262	Signal	0001692_	800	43	0.0497	0.109	1140	5.19E-05	Signal	0.00505	0.0052	0.0051	0.0001	M0+S+HIV	0.00514		1.676288
20	13.6	16.4	1085	0.00257	Signal	0001692_	800	44	0.051	0.114	1200	5.22E-05	Signal	0.00695	0.0070	0.0070	0.0000	M1+S+HIV	0.00698		2.489035
21	8.82	11.8	986	0.00299	Signal	0001692_	800	45	0.0505	0.113	1209	5.14E-05	Signal	0.00958	0.0096	0.0096	0.0000	M2+S+HIV	0.00958		3.271632
22	3.94	6.4	910	0.00271	Signal	0001692_	800	46	0.0334	0.0906	1102	5.19E-05	Signal	0.01416	0.0131	0.0136	0.0007	M0+R+S+H	0.01363		4.443268
23	26.5	29.6	1260	0.00249	Signal	0001692_	800	47	0.081	0.158	1344	5.71E-05	Signal	0.00534	0.0055	0.0054	0.0001	M1+R+S+H	0.00542		1.931691
24	14.5	17.9	1591	0.00211	Signal	0001692_	800	48	0.0469	0.114	1353	4.95E-05	Signal	0.00637	0.0063	0.0063	0.0001	M2+R+S+H	0.00633		2.162345

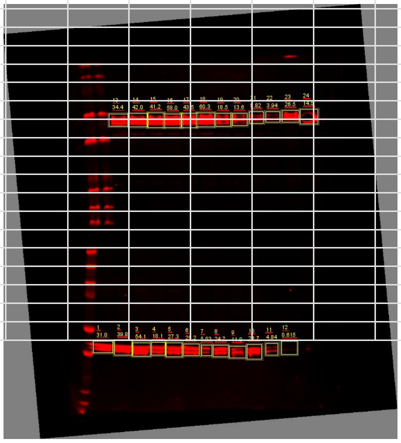

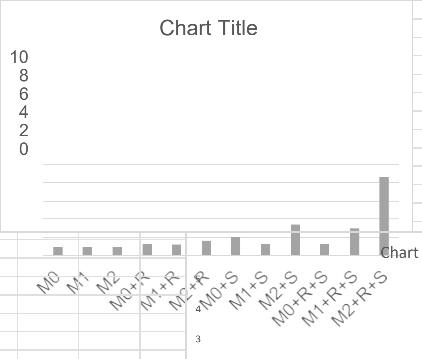

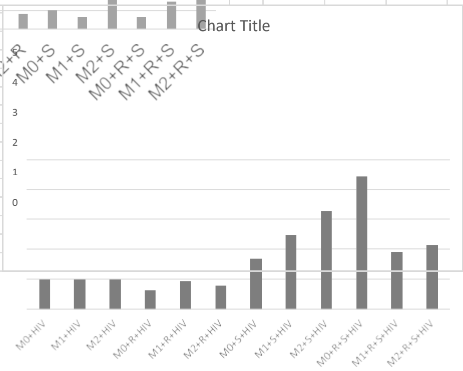

Group	Sample name	absorbanc	concentration (µg/mL)	Sample µ	Dye µl	lysis buffe
Add Res and Sirtinol and tinfectd with HIV	#1, M0_HIV	0.271	1648	24.3	15	20.7
	#2, M1_HIV	0.2334	1272	31.4	15	13.6
	#3, M2_HIV	0.2516	1454	27.5	15	17.5
	#4, M0_None	0.2199	1137	35.2	15	9.8
	#5, M1_None	0.2139	1077	37.1	15	7.9
	#6, M2_None	0.205	988	40.5	15	4.5
	#7, M0_Res	0.1759	697	57.4	15	-12.4
	#8, M1_Res	0.1885	823	48.6	15	-3.6
	#9, M2_Res	0.2008	946	42.3	15	2.7
	#10, M0_Sirtinol	0.1931	869	46.0	15	-1.0
	#11, M1_Sirtinol	0.2643	1581	25.3	15	19.7
	#12, M2_Sirtinol	0.1984	922	43.4	15	1.6
	#13, M0_HIV+Res	0.2157	1095	36.5	15	8.5
	#14, M1_HIV+Res	0.2216	1154	34.7	15	10.3
	#15, M2_HIV+Res	0.2234	1172	34.1	15	10.9
	#16, M0_HIV+Sirtinol	0.2261	1199	33.4	15	11.6
	#17, M1_HIV+Sirtinol	0.2399	1337	29.9	15	15.1
	#18, M2_HIV+Sirtinol	0.2911	1849	21.6	15	23.4
	#19, M0_HIV+Res+Sirtinol	0.2518	1456	27.5	15	17.5
	#20, M1_HIV+Res+Sirtinol	0.1508	446	89.7	15	-44.7
	#21, M2_HIV+Res+Sirtinol	0.1971	909	44.0	15	1.0
	#22, M0_Res+Sirtinol	0.1206	144	277.8	15	-232.8
	#23, M1_Res+Sirtinol	0.1396	334	119.8	15	-74.8
	#24, M2_Res+Sirtinol	0.1198	136	294.1	15	-249.1
Infected with HIV for 3d then add drugs for 2d	#25, M0_HIV	0.2515	1453	27.5	15	17.5
	#26, M1_HIV	0.2067	1005	39.8	15	5.2
	#27, M2_HIV	0.2585	1523	26.3	15	18.7
	#28, M0_None	0.1841	779	51.3	15	-6.3
	#29, M1_None	0.1705	643	62.2	15	-17.2
	#30, M2_None	0.174	678	59.0	15	-14.0
	#31, M0_Res	0.2322	1260	31.7	15	13.3
	#32, M1_Res	0.2302	1240	32.3	15	12.7
	#33, M2_Res	0.2012	950	42.1	15	2.9
	#34, M0_Sirtinol	0.2392	1330	30.1	15	14.9
	#35, M1_Sirtinol	0.2309	1247	32.1	15	12.9
	#36, M2_Sirtinol	0.2216	1154	34.7	15	10.3
	#37, M0_HIV+Res	0.2376	1314	30.4	15	14.6
	#38, M1_HIV+Res	0.2929	1867	21.4	15	23.6
	#39, M2_HIV+Res	0.2807	1745	22.9	15	22.1
	#40, M0_HIV+Sirtinol	0.2393	1331	30.1	15	14.9
	#41, M1_HIV+Sirtinol	0.2207	1145	34.9	15	10.1
	#42, M2_HIV+Sirtinol	0.2667	1605	24.9	15	20.1
	#43, M0_HIV+Res+Sirtinol	0.2454	1392	28.7	15	16.3
	#44, M1_HIV+Res+Sirtinol	0.1701	639	62.6	15	-17.6
	#45, M2_HIV+Res+Sirtinol	0.151	448	89.3	15	-44.3
	#46, M0_Res+Sirtinol	0.2155	1093	36.6	15	8.4
	#47, M1_Res+Sirtinol	0.2399	1337	29.9	15	15.1
	#48, M2_Res+Sirtinol	0.1999	937	42.7	15	2.3

Figure S5: Main text Figure 4: Histone acetylation raw data set and sample information.

Raw data sets for Figures 6A (CD4 and CD8 ratio):

Pre-challenge	15 dpi (HIV)/7 dpi (Mtb)	28 dpi (HIV)/21 dpi (Mtb)	35 dpi (HIV)/28 dpi (Mtb)
3.35	0.076852	0.245732	0.555085
3.16	0.11768	0.338776	0.438735
36		10.01346	8.587963
3.44	1.625	0.9	1.72
1.83	0.009634	0.358098	0.706004

Raw data for Figures 6B (HIV viral load):

Pre-challenge	15 dpi (HIV)/7 dpi (Mtb)	28 dpi (HIV)/21 dpi (Mtb)	35 dpi (HIV)/28 dpi (Mtb)
1	1	13137023.09	1.2E+08
1	1	1	1
1	1	1	1
1	1	30390881.28	1.93E+08
1	1	154940081.7	67910680

Raw data for Figures 6C (Lungs and spleen Mtb CFUs)

	Lung		Spleen	
Initial infection	Mtb Control	HIV-Mtb Co-infection	Mtb Control	HIV-Mtb Co-infection
50	124000	680000	5200	5400
30	560000	2000000	8600	41600
270	140000	208000	4600	40000
	86000	10400	3800	187200
	356000		15800	800
	160000		1200	
	736000		10000	
	396000		10000	
	466000		12000	

Raw data for Figures 6D (PET assay)

Rrs			Crs			Ers			Lung volume		
Uninfected	MtbControl	Mtbcoinfected	Uninfected	Mtbcontrol	Mtbcoinfected	Uninfected	Mtbcontrol	Mtbcoinfected	Uninfected	Mtbcontrol	Mtbcoinfected
0.869	2.282222	3.745	0.056	0.171306	0.011	17.914	5.839117	88.718	1260	711.33	637.7
0.78	7.870395	0.972	0.053	0.024731	0.036	18.884	40.43537	27.515	1290	1300	934.51
0.791	2.245803	1.182	0.055	0.012605	0.048	18.03	79.33481	20.721	1310	830.6	1070
	1.089791	0.897		0.032	0.047		31.411	21.079		1290	1190
	1.742787	0.881		0.014918	0.047		67.04054	21.338		1040	1170
	0.913262	3.642081		0.037181	0.011638		26.8992	86.05211		1510	1270
	1.088283	0.97224		0.032174	0.036345		31.08131	27.51465		1010	1110
		1.181838			0.048261			20.72088			1060
		0.896671			0.047441			21.07897			1150
		0.881023			0.046865			21.33839			

Figure S6: Raw data sets for Figure S6A-D.
